# Conducting linear chains of sulphur inside carbon nanotubes

**DOI:** 10.1038/ncomms3162

**Published:** 2013-07-12

**Authors:** Toshihiko Fujimori, Aarón Morelos-Gómez, Zhen Zhu, Hiroyuki Muramatsu, Ryusuke Futamura, Koki Urita, Mauricio Terrones, Takuya Hayashi, Morinobu Endo, Sang Young Hong, Young Chul Choi, David Tománek, Katsumi Kaneko

**Affiliations:** 1 Research Center for Exotic Nanocarbons (JST), Shinshu University, 4-17-1 Wakasato, Nagano 380-8553, Japan; 2 Institute of Carbon Science and Technology, Shinshu University, 4-17-1 Wakasato, Nagano 380-8553, Japan; 3 Department of Physics and Astronomy, Michigan State University, East Lansing, Michigan 48824, USA; 4 Department of Materials Science and Technology, Nagaoka University of Technology, 1603-1, Kamitomioka, Nagaoka 940-2188, Japan; 5 Department of Applied Chemistry, Faculty of Engineering, Nagasaki University, 1-14 Bunkyo-machi, Nagasaki-shi, Nagasaki 852-8521, Japan; 6 Department of Physics, Department of Chemistry, Department of Material Science and Engineering and Center for 2-Dimensional and Layered Materials, The Pennsylvania State University, University Park, Pennsylvania 16802, USA; 7 Faculty of Engineering, Shinshu University, 4-17-1 Wakasato, Nagano 380-8553, Japan; 8 CNT Team, Hanwha Chemical Corporation, 80 Annamro 402 gil, Bupyeong-gu, Incheon 403-030, Republic of Korea

## Abstract

Despite extensive research for more than 200 years, the experimental isolation of monatomic sulphur chains, which are believed to exhibit a conducting character, has eluded scientists. Here we report the synthesis of a previously unobserved composite material of elemental sulphur, consisting of monatomic chains stabilized in the constraining volume of a carbon nanotube. This one-dimensional phase is confirmed by high-resolution transmission electron microscopy and synchrotron X-ray diffraction. Interestingly, these one-dimensional sulphur chains exhibit long domain sizes of up to 160 nm and high thermal stability (~800 K). Synchrotron X-ray diffraction shows a sharp structural transition of the one-dimensional sulphur occurring at ~450–650 K. Our observations, and corresponding electronic structure and quantum transport calculations, indicate the conducting character of the one-dimensional sulphur chains under ambient pressure. This is in stark contrast to bulk sulphur that needs ultrahigh pressures exceeding ~90 GPa to become metallic.

## Abstract (web summary)

Elemental sulphur is an insulator in the bulk phase, although it may become conducting under ultrahigh-pressure conditions. Here, the authors report a one-dimensional conducting form of sulphur formed by encapsulation inside single-walled and double-walled carbon nanotubes.

## Graphical abstract

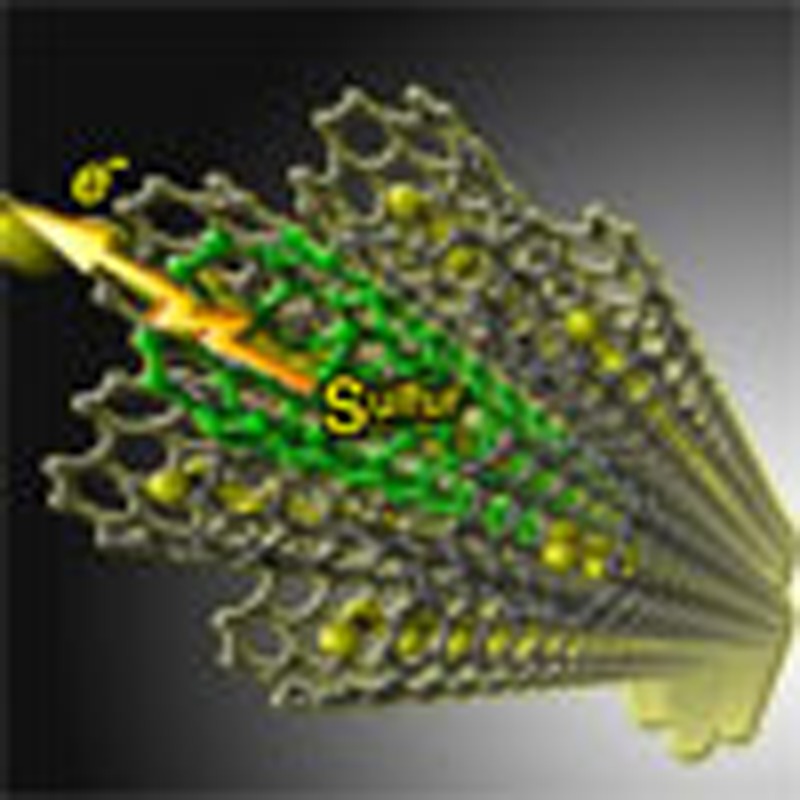

Elemental sulphur, the most polymorphic element, is an insulator in the bulk three-dimensional (3D) structure under ambient conditions. Only at ultrahigh pressures exceeding ~90 GPa, does it undergo a structural phase transition to a base-centred orthorhombic metallic phase^1^,^2^,^3^,^4^. From this base-centred orthorhombic phase, it is possible to hypothetically extract a one-dimensional (1D) sulphur chain exhibiting a zigzag configuration. This 1D system has been predicted to show a finite electronic density of states near the Fermi level and thus to display metallic behaviour^5^,^6^,^7^. Furthermore, Springborg and Jones^5^,^6^ have proposed that the metallic behaviour of sulphur could also result from a free-standing 1D linear configuration. Despite the fact that 3D sulphur behaves as an insulator^1^, to the best of our knowledge, the experimental isolation of 1D sulphur with metallic properties has not been reported so far, because it is almost impossible to preserve 1D chains without any supports.

A common problem found in free-standing 1D metallic systems is the Peierls instability associated with bond length alternation, which opens a fundamental energy gap and leads to a metal-insulator transition^8^: 1D conducting polymers (for example, polyacetylene) constitute a well-known 1D system undergoing Peierls transition^9^. A different way to suppress the Peierls transition in 1D metallic systems that form zigzag or other nonlinear chains is to sterically constrain the nonlinear structure inside a narrow cylindrical cavity. Even small lattice distortions of the enclosed 1D metal may change the electronic structure near the Fermi level^10^ sufficiently to suppress the Peierls instability and preserve the enclosed nanowire as a 1D quantum conductor.

In this context, single-walled carbon nanotubes (SWCNTs) turn out to be superior host materials in comparison with other porous materials because of the presence of closed quasi-1D channels with diameter of ≈1 nm (refs 11, 12, 13, 14, 15, 16, 17, 18, 19). In addition, SWCNTs exhibit unique 1D electronic behaviour ranging from metallic to semiconducting, depending on the nanotube chirality (given by the diameter and the orientation of the hexagonal honeycomb along the tubule axis)^20^. Furthermore, carbon nanotubes (CNTs) are highly rigid because of the stability of sp^2^ carbon bonds. This rigidity efficiently suppresses the Peierls instability in metallic SWCNTs (M-SWCNT)^21^. The steric constraints provided by the enclosing rigid SWCNT should therefore also suppress a potential Peierls instability in the enclosed 1D chain.

Here we report the successful synthesis of 1D chains of sulphur inside SWCNTs and double-walled CNTs (DWCNTs) using a vapour reaction method. The modified protocol resembles the well-established procedure used to encapsulate fullerenes inside SWCNTs^22^. We demonstrate the highly ordered 1D zigzag and linear sulphur chains with long domain sizes up to 160 nm and high thermal stability (~800 K). Most importantly, the 1D sulphur chains exhibit a conducting character under ambient pressure, which is also supported by *ab initio* density functional calculations.

## Results

### Structural characterization of 1D sulphur chains

**Figure 1:**
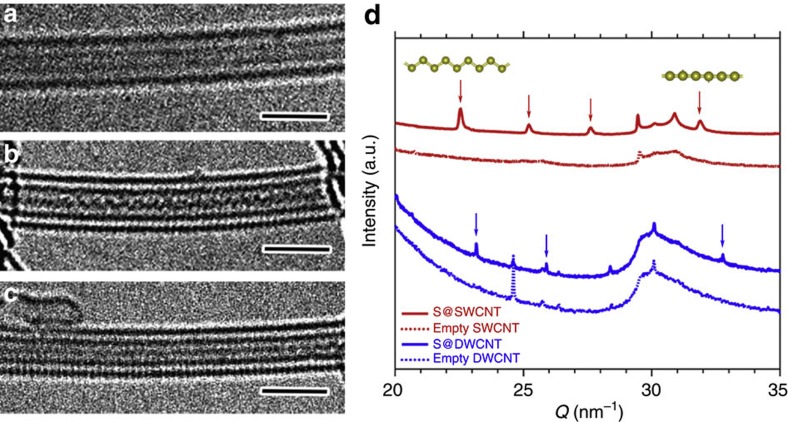
Structural identification of 1D sulphur chains inside CNTs. (**a**) HRTEM image of S@SWCNT. The two lines correspond to 1D sulphur chains encapsulated inside a SWCNT. (**b**) HRTEM image of S@DWCNT with the 1D sulphur chain in zigzag conformation. (**c**) A 1D linear chain inside a DWCNT. Scale bar, 2 nm. (**d**) XRD profiles of S@SWCNTs, empty SWCNTs, S@DWCNTs and empty DWCNTs. Arrows indicate the Bragg peaks of 1D sulphur chains.

Figure 1a displays high-resolution transmission electron microscopy (HRTEM) images of sulphur encapsulated inside a SWCNT (S@SWCNT). In these images, we can identify two lines separated by 0.32 nm inside the hollow core of a SWCNT with diameter of 1.1 nm, which we interpret as two parallel monatomic sulphur chains. Furthermore, those sulphur chains seem to move simultaneously along the tube axis, activated by electron-beam irradiation during the HRTEM observations (see Supplementary Fig. S1). Occasionally, the motion of the sulphur chains inside a SWCNT generated an arrangement with one chain obscuring the other in the HRTEM image. The surrounding CNT walls allowed us to identify the encapsulated structure with atomic resolution by stabilizing it in place and also by protecting it from electron-beam damage during imaging. For this reason, a clear 1D zigzag atomic chain of sulphur could be identified by HRTEM inside a DWCNT (S@DWCNT) with an inner diameter of 0.7 nm, as depicted in Fig. 1b. A different linear sulphur chain was also observed inside a DWNT with a narrower inner diameter of 0.6 nm (Fig. 1c). Our HRTEM analysis reveals that the mean periodic distance in axial direction is 0.33±0.03 nm for the zigzag chains and 0.18±0.02 nm for the linear sulphur chains. From a theoretical standpoint, as the number of sulphur atoms per primitive unit cell is reduced from two to one during the zigzag-to-linear transition, we should compare supercells with two sulphur atoms, independent of conformation. In this case, the periodic distance of the two-atom supercell of the linear chain is 0.36±0.04 nm, larger than that of the zigzag chain. This agrees with our expectation that with decreasing nanotube diameter, the constrained sulphur chains generally become straighter, and their periodic distance in axial direction increases (see Supplementary Fig. S2).

Our X-ray diffraction (XRD) analysis reveals the presence of highly ordered zigzag or linear atomic sulphur chains (Fig. 1d). The XRD pattern of S@SWCNTs clearly shows asymmetric lambda-shaped Bragg peaks at the scattering vectors *Q*=22.5, 25.2, 27.6 and 31.9 nm^−1^, which are characteristic of a 1D structure^14^,^23^,^24^,^25^. These *Q*-values correspond to 1D lattice constants *d* ranging from *d*=0.197 to 0.279 nm. This particular distribution of lattice constants reflects the different degrees of distortion, to which the enclosed sulphur chains are subjected to by their surrounding nanotubes with a particular diameter distribution. Different lattice constants may occur in a single sulphur chain inside a defective CNT that may change its diameter or pin down sulphur atoms at wall defects. The shortest 0.197 nm spacing is only a little shorter than the bond length in other sulphur allotropes (for example, 0.2046, nm in α-S_8_ ring molecule, 0.2066, nm in fibrous sulphur S_ψ_)^1^. We thus associate *d*=0.197 nm with a linear chain and *d*=0.279 nm with a zigzag chain encapsulated inside a SWCNT. The 1D sulphur chains inside DWCNTs also exhibit zigzag and linear conformations, as observed in HRTEM images shown in Fig. 1b. The axial lattice constant values ranging from *d*=0.192 to 0.271 nm were slightly shorter than inside SWCNTs. Also, these *d* values show good agreement with the HRTEM analysis, which reveals the mean periodic distances to be 0.18±0.02 nm in linear and 0.33±0.03 nm in zigzag sulphur chains (see Supplementary Fig. S2). Therefore, the 1D sulphur chains encapsulated inside SWCNTs and DWCNTs are very similar, except for a slight axial compression inside the DWCNTs.

The domain size of the 1D chains of sulphur, *ξ*, has a crucial role in electric transport properties, because ends of short sulphur chain segments act as scattering centres and alter electron transport of the entire system. The domain size is estimated using *ξ*=2*π*/Δ*Q,* where Δ*Q* is the full width at half maximum of the corresponding Bragg peak^14^. In particular, the domain size of the 1D sulphur chains inside SWCNTs is 35–45 nm, and that within DWCNTs is 90–160 nm at 300 K. Assuming that the typical length of a CNT is ~1 μm, S@SWCNTs contain ~20–30 domains and S@DWCNTs contain ~6–10 domains of 1D sulphur chains. We interpret this long-range order and large domain size as a consequence of the strong covalent bond character within the 1D sulphur chains.

**Figure 2:**
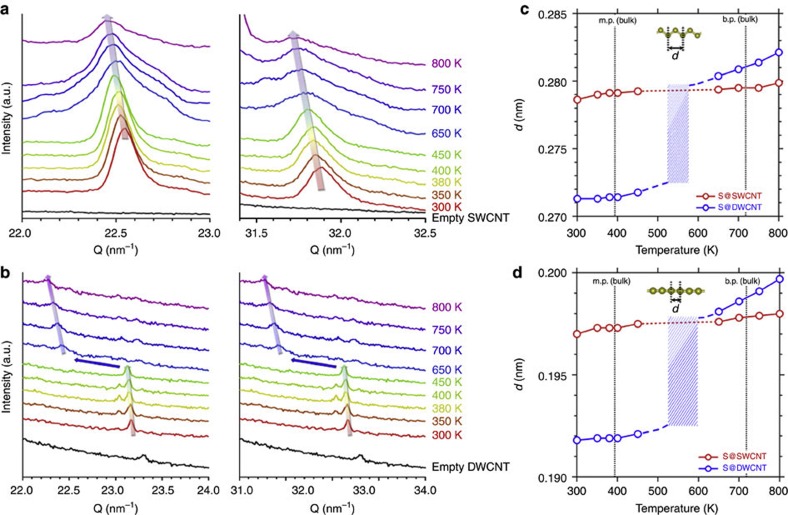
Thermal stability of 1D sulphur chains with zigzag and linear geometries. Temperature dependence of the 1D Bragg peaks of sulphur inside (**a**) SWCNTs and (**b**) DWCNTs. Temperature dependence of the axial lattice constant *d* in (**c**) 1D zigzag and (**d**) linear sulphur chains. The melting point (m.p.) and boiling point (b.p.) of bulk sulphur (α-S_8_) are shown for comparison. A sharp increase in the *d* value of S@DWCNT between 450–650 K indicates a structural change of the 1D chains.

We further tested the temperature dependence of the 1D Bragg peaks to assess the thermal stability and phase transitions of the 1D sulphur chains in the zigzag and linear geometries. For S@SWCNTs consisting of two parallel 1D chains of enclosed sulphur, the *Q*-values of Bragg peaks assigned to zigzag and linear lattices decrease slightly as the temperature increases up to 800 K (Fig. 2a). This thermal contraction, driven by configurational entropy, is rather common in polymers. Though Δ*Q* values become broad at temperatures higher than 650 K, the 1D Bragg peaks are still pronounced at 800 K, indicating a high thermal stability of the 1D double chains of sulphur. For DWCNTs encapsulating single 1D chains in the inner tube, we found an abrupt decrease in the *Q*-value of the peak at *T*≈650 K (Fig. 2b), implying a large structural change in this 1D system. Interestingly, the lattice constants of the 1D chains in S@DWCNTs closely approach the values found in S@SWCNT >650 K (Fig. 2c). This result indicates that SWCNTs constitute the most suitable cavities for stabilizing 1D chains of sulphur. In comparison with bulk arrays of S_8_ ring molecules (the most stable sulphur allotrope with melting point at ~393 K and boiling point at ~718 K), 1D sulphur chains in both S@SWCNT and S@DWCNT display very different thermodynamic behaviour. Very different from the bulk sulphur phase, which undergoes complicated crystallographic and structural transitions^1^,^26^ including 3D trigonal and tetragonal chains at high pressures^27^,^28^,^29^, sulphur chains are immobilized in the host CNTs up to 800 K.

**Figure 3:**
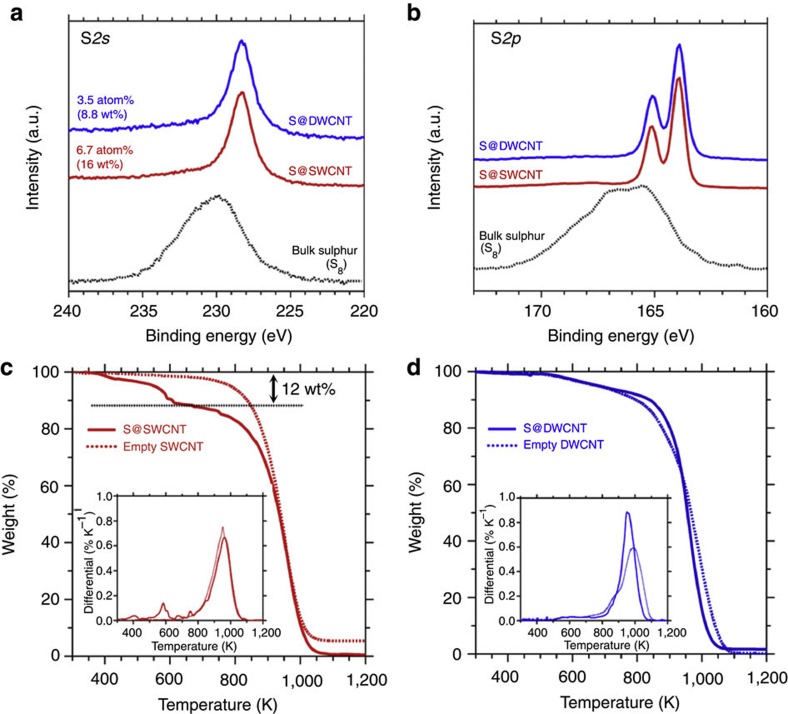
Chemical states and filling ratios of 1D sulphur chains. XPS spectra in the (**a**) S*2s* and (**b**) S*2p* core-level regions of empty SWCNTs, S@SWCNTs, empty DWCNTs and S@DWCNTs, in comparison with bulk sulphur (α-S_8_). Filling ratios of the 1D sulphur chains, evaluated by XPS analysis, are 6.7 atom% (16 wt%) for S@SWCNTs and 3.5 atom% (8.8 wt%) for S@DWCNTs. TG curves of (**c**) empty SWCNTs and S@SWCNTs and (**d**) empty DWCNTs and S@DWCNTs, measured under He/O_2_ gas flow (He:O_2_=8:2). Insets show the differential TG curves. TG analysis reveals that the filling ratio is 12 wt% for S@SWCNTs, in good agreement with values obtained by XPS. As the oxidation reactions start at nearly the same temperature for DWCNTs and 1D chains of sulphur encapsulated in DWCNTs (~800 K), the filling ratio of S@DWCNTs could not be evaluated by TG analysis.

To understand the chemical bonding of 1D sulphur chains, we performed X-ray photoelectron spectroscopy (XPS) studies in the S*2s* and S*2p* core-level regions (Fig. 3a) and resonance Raman spectroscopy in the frequency range where the characteristic conformation of S–S bonding could be extracted for the 3D bulk systems (see Supplementary Fig. S3). The XPS S*2s* and S*2p* spectra of S@SWCNTs and S@DWCNTs look very similar, indicating that the chemical bonding of sulphur atoms in the chains is identical, as already suggested by the crystallographic information extracted from the XRD analysis. As seen in Fig. 3a, the S*2s* and S*2p* core-level binding energies of the 1D sulphur chains are smaller than those of the bulk S_8_ ring molecule (see Supplementary Table S1, Supplementary Fig. S4 and Supplementary Note 1 for details). The lower binding energies reflect the strain, to which sulphur chains enclosed inside CNTs are subjected. Unlike the bulk 3D sulphur^30^, the S–S stretching modes are obscured in resonance Raman spectra of the strained sulphur chains stabilized inside CNTs (see Supplementary Fig. S3), implying that the encapsulated sulphur chains exhibit a 1D planar conformation. It should also be noted that the XPS C*1s* core-level spectra of the enclosing CNTs (see Supplementary Fig. S5 and Supplementary Note 2) are nearly unaffected by the presence of enclosed sulphur chains. This indicates that there is no or only a negligible chemical interaction between the sulphur chains and the host CNTs. The absence of C*1s* core-level shifts due to the presence of sulphur also indicates that the charge transfer between the nanotubes and sulphur chains should be negligible. This agrees with the computational results presented below and can be rationalized by the fact that the electronegativity of sulphur and carbon has the same value of 2.5 on the Pauling scale^31^.

The intensity analysis of our XPS spectra indicates that an atomic filling ratio of sulphur is 6.7 atom% (16 wt%) for S@SWCNT (Fig. 3a). This is in good agreement with the thermogravimetric (TG) analysis obtained during combustion, indicating 12 wt% of filled sulphur, as seen in Fig. 3c. Our XPS analysis of S@DWCNT indicates a lower S filling ratio of 3.5 atom% (8.8 wt%). This quantity could not be verified by TG analysis, because the combustion temperature of DWCNTs and sulphur is the same (Fig. 3d). These high atomic filling ratios, together with the results of our elemental mapping analysis that find coinciding spatial distribution of carbon and sulphur (see Supplementary Figs S6 and S7 and Supplementary Note 3), indicate that sulphur is uniformly distributed inside the CNTs.

### Conducting character of 1D sulphur chains

**Figure 4:**
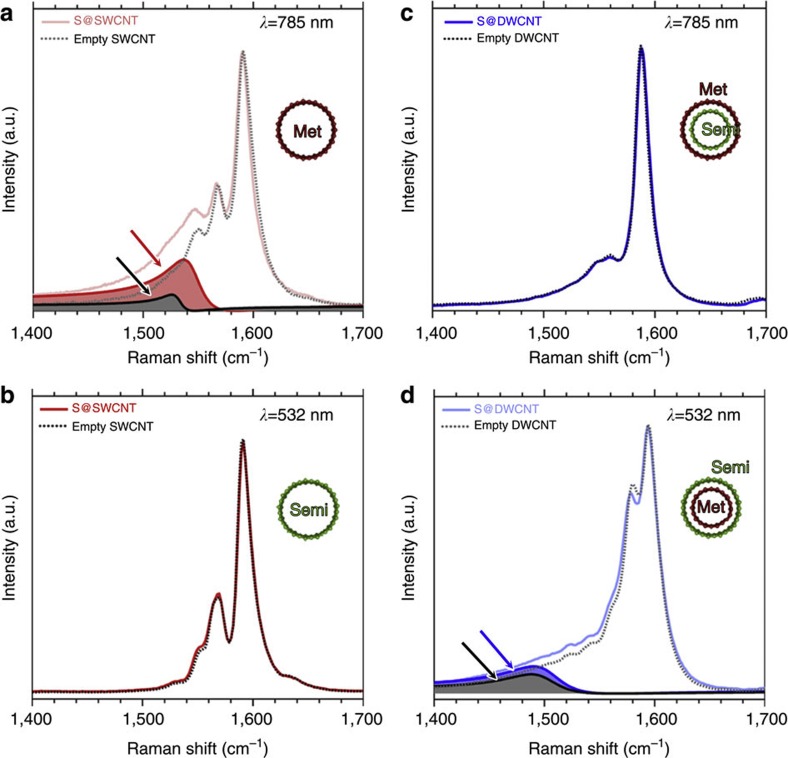
Spectroscopic evidence for metallicity of 1D sulphur chains. Resonance Raman spectra of S@SWCNTs in comparison with empty SWCNTs, measured with 785 nm (**a**) and 532 nm (**b**) laser excitations. Raman spectra of empty DWCNTs and S@DWCNTs, measured with 785 nm (**c**) and 532 nm (**d**) laser excitations. The asymmetric BWF lines associated with metallic CNTs or enhanced in presence of metallic 1D sulphur chains are highlighted by the arrows in **a** and **d**. Met, metallic CNT; Semi, semiconducting CNT.

Raman spectroscopy not only probes vibration spectra but also can be used to extract information about metallicity of the 1D sulphur chains. Specifically, an asymmetric Raman signal in the low-frequency shoulder of the tangential G^+^-mode at ~1590, cm^−1^, the so-called Breit–Wigner–Fano (BWF) line, is associated with metallic character in graphitic materials including M-SWCNTs^32^,^33^,^34^,^35^. Figure 4 compares the Raman spectra of S@SWCNTs and S@DWCNTs with those of empty CNTs. We chose two different excitation wavelengths, *λ*=532 and 785 nm, to selectively probe either semiconducting or metallic CNTs under resonant Raman conditions, which depend on the electronic structure of the corresponding CNTs^36^. Among our SWCNT samples, M-SWCNTs represent the main component in the spectrum obtained using the 785 nm laser excitation, whereas semiconducting SWCNTs dominate the 532 nm laser excitation spectrum (see Supplementary Figs S8 and S9). The BWF line turns out to be more intense in the presence of encapsulated 1D sulphur chains within M-SWCNTs (Fig. 4a), thus implying that the presence of sulphur enhances the electronic conduction of CNTs. This result provides evidence that sulphur chains constrained in the nanotube enclosure are metallic (see Supplementary Note 4), thus enhancing the electrical conductivity of the carbon-sulphur hybrid system.

To learn more about specific conditions of sulphur-related conductivity enhancement, we compared the BWF lines in Raman spectra of metallic and semiconducting CNTs enclosing sulphur chains in Fig. 4. Unlike sulphur in a M-SWCNT shown in Fig. 4a, no BWF resonance occurs because of the presence of sulphur in a semiconducting SWCNT (Fig. 4b). Moreover, in a DWCNT with a metallic outer tube in resonance with the 785 nm laser excitation (see Supplementary Fig. S10) and a semiconducting inner tube, the presence of sulphur does not enhance the intensity of the BWF line in the outer wall (Fig. 4c). For a DWCNT with a semiconducting outer tube and a metallic inner tube in resonance with the 532 nm laser excitation (see Supplementary Fig. S11), the BWF line is slightly enhanced in the presence of encapsulated 1D sulphur chains (Fig. 4d). These results suggest a direct contact between the metallic 1D sulphur chains and the metallic character of the enclosing CNTs, which seems to be the key for enhancing the electrical conductivity of hybrid carbon-sulphur systems.

Not only the Raman-active G^+^-mode but also the 2D mode (see Supplementary Table S2) reflect the interaction of enclosed sulphur chains with the enclosing wall in S@SWCNTs. We concluded that the presence of enclosed sulphur does not change the G^+^-band frequency, indicating the absence of strong chemical bonds or charge transfer in the system (see Supplementary Note 5). The absence of charge transfer had been confirmed independently by XPS spectra (see Supplementary Fig. S5). In contrast to the G^+^-mode, the 2D blue band shifts up to 9 cm^−1^ due to enclosed sulphur chains. Although such frequency shifts of the 2D mode may be caused by chemical doping of nanotubes^37^, the blue shift in this case is caused by the presence of the sulphur chain inside the nanotube which hinders the motion of carbon atoms and thus hardens the vibrational modes.

**Figure 5:**
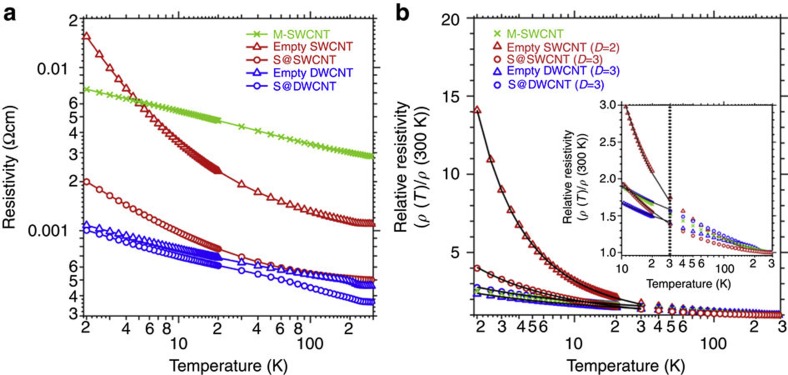
Electric transport of buckypapers containing 1D sulphur chains. (**a**) Resistivity of films (buckypapers) consisting of empty SWCNTs, S@SWCNTs, empty DWCNTs and S@DWCNTs as a function of temperature, in comparison with pristine M-SWCNTs. (**b**) Temperature dependence of the relative film resistivity, normalized by the *T=*300 K value, fitted with the variable range hopping model (black solid lines). *D* represents the dimensionality of electron hopping in these systems. The inset in **b** shows the magnified relative resistivity curves (10–300 K). The relative resistivity curve of M-SWCNTs could not be reproduced using the variable range hopping model^41^.

In order to verify that the presence of 1D sulphur chains indeed enhances the electron transport in nanotubes containing sulphur chains, we performed direct-current electrical resistance measurements for buckypapers containing empty and sulphur-filled metallic CNTs, as well as pristine M-SWCNTs, and report our findings in Fig. 5a. Our results indicate a significant drop in resistivity caused by encapsulation of 1D sulphur chains in SWCNTs. The temperature-dependent measurements indicate that the resistivity of all systems decreases with increasing temperature, which is characteristic of systems with a low density of states at *E*_F_. At 300 K, we observe that the electric resistivity *ρ* (300 K) of SWCNTs drops by one half from 1.1 × 10^−3^ to 5.0 × 10^−4^ Ωcm. At 2 K, the resistivity values for pristine SWCNTs corresponding to *ρ* (2 K)=1.5 × 10^−2^ Ωcm drop by a whole order of magnitude to 2.0 × 10^−3^ Ωcm because of the presence of enclosed sulphur chains. The same general trends, but even with lower resistivity values across the whole temperature range, occur in pristine and S-filled DWCNT systems when compared to the equivalent SWCNT systems. We need to point out that resistivity measurements in buckypapers are strongly affected by the morphology of the sample, which consist of aggregates of entangled CNT bundles of different length connected at many unspecific contact points. The observed low resistivity of M-SWCNTs in comparison with other empty CNTs, may be caused not only by their intrinsic metallic character but also by their shorter average length, a consequence of the dispersion/separation process^38^,^39^ that affects the buckypaper morphology. Assuming that the contact resistance within the buckypaper is temperature independent, we normalized the resistivity *ρ* (*T*) at a given temperature by *ρ* (300 K) to suppress the effect of different contact morphologies and plotted the temperature-dependent relative resistivity *ρ* (*T*)/*ρ* (300 K) in Fig. 5b. As suggested earlier, also the relative resistivity of S@SWCNTs is lower than that of empty SWCNTs, indicating additional electron transport channels present in the hybrid system.

We modelled the observed conductance data by the variable range hopping model^40^,^41^,^42^,^43^ to evaluate the dimensionality of percolation paths due to electron hopping. We found the relative resistivity curve *ρ* (*T*)/*ρ* (300 K) of S@SWCNTs to be well reproduced by assuming 3D hopping processes, whereas that of empty SWCNTs follows a 2D hopping behaviour. The corresponding fits, shown by the black lines in Fig. 5b, indicate that the 1D sulphur chains establish new conduction paths throughout the system. We find the relative resistivity of S@SWCNT to be comparable to that of M-SWCNTs >10 K, displaying the lowest resistivity values among the samples.

### *Ab initio* quantum conductance study of 1D sulphur chains

**Figure 6:**
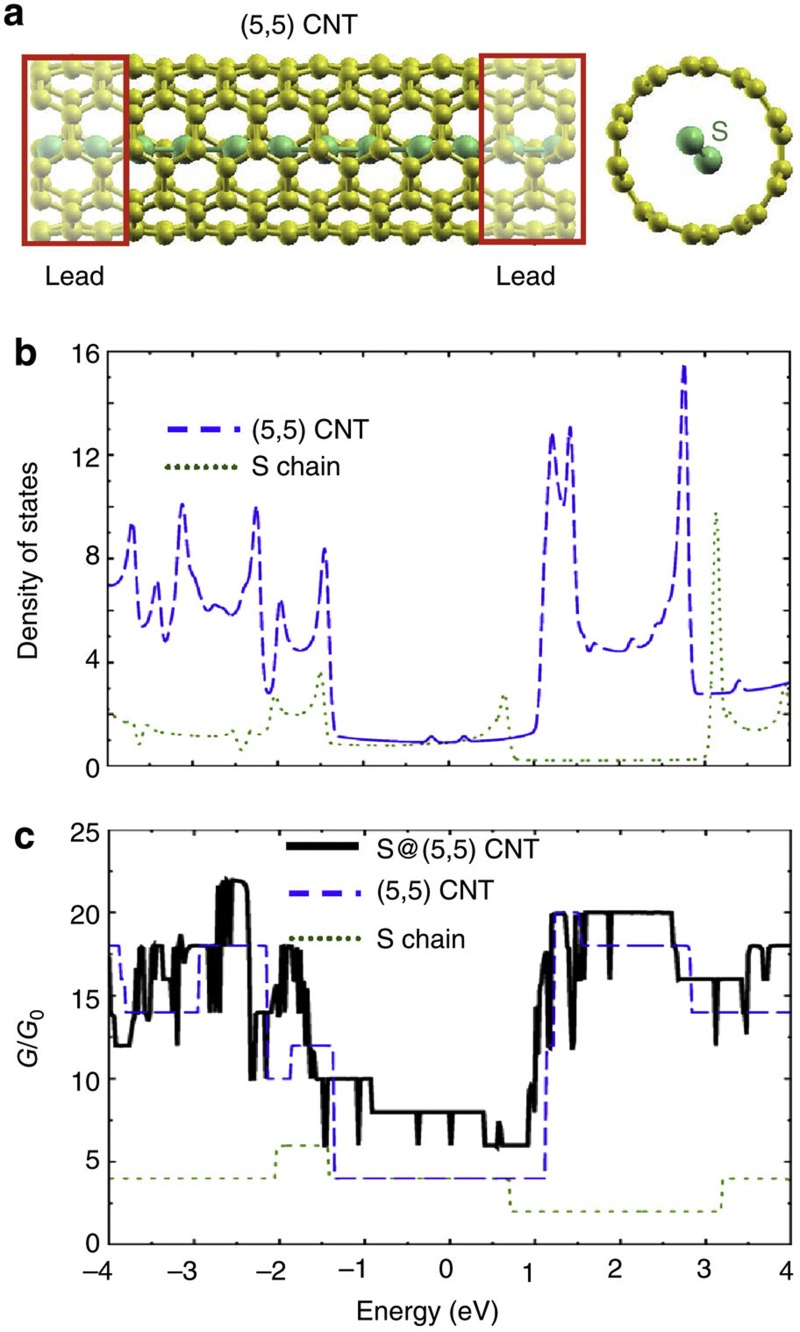
Electronic properties of a sulphur chain inside a CNT. (**a**) Side and end-on view of a sulphur chain encapsulated inside a (5,5) SWCNT. (**b**) Electronic density of states of a (5,5) SWCNT and an isolated sulphur chain. (**c**) Ballistic quantum conductance of a sulphur chain encapsulated inside a (5,5) SWCNT along with that of an isolated SWCNT and an isolated sulphur chain. *E*=0 denotes the Fermi level in **b** and the carrier injection energy in a neutral, unbiased system in **c**. *G*_0_ is the conductance quantum.

To better understand the stability and electronic properties of these novel hybrid systems, we performed *ab initio* density functional calculations of isolated sulphur chains and sulphur chains contained inside the inner core of (5,5) and (8,0) CNTs (see Supplementary Figs S12–S15). Our results for the quantum conductance of a sulphur chain encapsulated inside a (5,5) CNT are presented in Fig. 6. The sulphur chain and the CNT are both metallic, because their electronic densities of states are nonzero at the Fermi level (see Fig. 6b). As the density of states of S@CNT in Fig. 6b is close to the superposition of that of a neutral nanotube and a sulphur chain, we conclude that there is no significant bonding or charge transfer between the chain and the nanotube. Our Mulliken population analysis indicates a negligible charge transfer of only ~0.1 electrons/sulphur atom between sulphur and the surrounding CNT (see Supplementary Note 6). The quantum conductance of the S@SWCNT system, as well as its components, calculated using the scattering geometry illustrated in Fig. 6a, is shown in Fig. 6c. As mentioned above, the electronic interaction between the encapsulated sulphur chain and the enclosing nanotube is very weak. Then, the total conductance of the S@SWCNT system should be close to the superposition of the quantum conductances of the isolated chain and the isolated nanotube. These quantum conductances are plotted in Fig. 6c, together with the conductance of the S@SWCNT. In each case, as indicated in Fig. 6a, we used the same system to describe the leads and the scattering region. With *G=*8*G*_0_, we find the quantum conductance of the unbiased and neutral S@SWCNT system to be twice as high as the M-SWCNT. Even at other injection energies, the conductance of the S@SWCNT system lies very close to the superposition of the isolated sulphur chain and the SWCNT, with the exception of dips in the conductance curve that are characteristic of the small sulphur–SWCNT interaction. Thus, our calculations provide solid support for the conclusion that sulphur chains do not modify the electronic conductance of CNTs, but rather introduce new conduction channels in the S@CNT system.

## Discussion

We have experimentally identified the presence of conducting 1D zigzag and linear sulphur chains, which self-assemble in the narrow cylindrical cavity inside SWCNTs and DWCNTs. Our XRD analysis reveals the long-range order and large domain size of the 1D sulphur chains inside CNTs at 300 K, resulting from the strong covalent bond in the 1D sulphur chains. Because of the covalent bonding character, we found high thermal stability of the 1D sulphur chains inside CNTs. The 1D sulphur chains retain those 1D structures up to 800 K of which temperature is much higher than boiling point of the bulk S_8_. Those results, as also supported by negligible charge transfer between 1D sulphur chains and CNTs, lead us to believe that the 1D sulphur chains act as a previously not observed conducting polymer consisting only of elemental sulphur, thus improving the electrical conductivity of CNTs.

The identification of a metallic phase of sulphur at ambient pressures is highly noteworthy, because bulk sulphur requires very high pressures >90 GPa to become metallic. We expect that the present results for CNT enclosures may be realized also in other nanoporous systems, including covalent organic frameworks^44^ and zeolites^45^, thus opening new avenues to study the chemistry of sulphur at the nanoscale. Since the electronic structure changes under steric constraints are not limited to sulphur, we foresee a new branch of chemistry evolving in the area of artificial stabilization of exotic structures inside nanoporous systems.

## Methods

### Synthesis of 1D chains of sulphur

Reagents were obtained from commercial suppliers and used without further purification. The SWCNT sample produced by the arc-discharged method was provided by Hanwha Nanotech, and the DWCNT sample was purchased from Toray Industries, Inc. The sorted sample of M-SWCNTs (99%) was purchased from NanoIntegris Inc. and used after annealing treatment at 773 K under 1 Pa for 1 h. High-purity (99.9999%), bright-yellow crystal of sulphur was used for avoiding contaminants. To encapsulate sulphur in the CNTs efficiently, the terminating caps on the SWCNT and DWCNT samples were first removed by oxidation treatment at 723 K under dry air (100 ml min^−1^) for 1 h. The open-ended CNT samples and the crystalline sulphur were sealed in a forked glass tube *in vacuo* (<1 Pa) and subsequently kept at 873 K for 48 h. The as-prepared sample was then washed with carbon disulphide to dissolve excess sulphur attached to the outside of the CNTs. Finally, S@CNT sheets (buckypaper) were obtained after filtration of the S@CNT-carbon disulphide solution and subsequent drying at 383 K *in vacuo* (<1 Pa).

### Analytical techniques

HRTEM observations were carried out with a double Cs-corrected (CEOS GmbH) HRTEM (JEM-2100F; JEOL) operated at 80 kV. XRD profiles were measured using the synchrotron X-ray source at SPring-8 (*λ*=0.08003, nm). The samples were sealed in a glass capillary *in vacuo* (<1 Pa). Temperature was controlled using an N_2_ gas injection gun. XPS measurements were performed using a monochromatized AlKα X-ray source (AXIS-ULTRA DLD; Shimazu). TG measurements were carried out under He/O_2_ (8:2) gas flow in the temperature range 300–1,200 K at a scan rate of 3 K min^−1^ (Thermo-Mass; Rigaku Corporation). The flow rate was kept at 300 ml min^−1^. Elemental mapping analyses were performed by energy-dispersive spectroscopy. The spectroscope (JED-2300T; JEOL) was coupled to a scanning transmission electron microscope operated at 120 keV. Raman spectra were obtained with 532 and 785 nm excitations, using a single-monochromator micro-Raman spectrometer in back scattering configuration (inVia Raman Microscope; Renishaw). Direct-current electric-resistance measurements were performed by the four-probe method, using a physical properties measurement system (model 6000; Quantum Design) in the temperature range from room temperature to 2 K. Transport measurements were performed on the S@CNT sheet (buckypaper).

### Computational methods

Our calculations of the equilibrium structure, stability and electronic properties of sulphur chains inside SWCNTs have been performed using *ab initio* density functional theory, as implemented in the SIESTA code^46^. We used the spin-polarized Ceperley–Alder^47^ exchange-correlation functional as parameterized by Perdew and Zunger^48^, norm-conserving Troullier–Martins pseudopotentials^49^ and a double-ζ basis including polarization orbitals. We used periodic boundary conditions to represent arrays of aligned, but well separated, SWCNTs containing sulphur. The Brillouin zone of the isolated 1D chain and nanotube structures was sampled by at least the equivalent of 16 *k*-points. We used a mesh cutoff energy of 180 Ry to determine the self-consistent charge density, which provided us with a precision in total energy of <7 meV per atom. Quantum transport calculations were performed using the TRANSIESTA code^50^, based on the non-equilibrium Green’s function approach.

## Author contributions

T.F. conceived the project. T.F., M.T. and D.T. prepared the manuscript. T.F., A.M.-G., H.M., K.U., and T.H. carried out the experiments. R.F. supported interpretation of the XRD data. Z.Z. performed DFT and transport calculations. S.Y.H. and Y.C.C. produced the SWCNT sample. M.T., M.E., D.T. and K.K. supervised the research.

## Additional information

**How to cite this article:** Fujimori, T. *et al.* Conducting linear chains of sulphur inside carbon nanotubes. *Nat. Commun.* 4:2162 doi: 10.1038/ncomms3162 (2013).

## Supplementary Material

Supplementary Information Supplementary Figures S1-S15, Supplementary Tables S1-S2, Supplementary Notes 1-6 and Supplementary References
