# Publisher Correction: Conducting linear chains of sulphur inside carbon nanotubes

**DOI:** 10.1038/s41467-022-28704-y

**Published:** 2022-02-28

**Authors:** Toshihiko Fujimori, Aarón Morelos-Gómez, Zhen Zhu, Hiroyuki Muramatsu, Ryusuke Futamura, Koki Urita, Mauricio Terrones, Takuya Hayashi, Morinobu Endo, Sang Young Hong, Young Chul Choi, David Tománek, Katsumi Kaneko

**Affiliations:** 1 Research Center for Exotic Nanocarbons (JST), Shinshu University, 4-17-1 Wakasato, Nagano, 380-8553 Japan; 2 Institute of Carbon Science and Technology, Shinshu University, 4-17-1 Wakasato, Nagano, 380-8553 Japan; 3 Department of Physics and Astronomy, Michigan State University, East Lansing, Michigan 48824 USA; 4 Department of Materials Science and Technology, Nagaoka University of Technology, 1603-1, Kamitomioka, Nagaoka, 940-2188 Japan; 5 Department of Applied Chemistry, Faculty of Engineering, Nagasaki University, 1-14 Bunkyo-machi, Nagasaki-shi, Nagasaki, 852-8521 Japan; 6 Department of Physics, Department of Chemistry, Department of Material Science and Engineering and Center for 2-Dimensional and Layered Materials, The Pennsylvania State University, University Park, Pennsylvania 16802 USA; 7 Faculty of Engineering, Shinshu University, 4-17-1 Wakasato, Nagano, 380-8553 Japan; 8 CNT Team, Hanwha Chemical Corporation, 80 Annamro 402 gil, Bupyeong-gu, Incheon, 403-030 Republic of Korea

Correction to: *Nature Communications* 10.1038/ncomms3162, published online 12 July 2013.

This Article contains an error in Fig. 2. The submitted and peer reviewed versions of this Article contained two distinct spectra in Fig. 2b, however one of those spectra was inadvertently duplicated during the production process. The correct version of Fig. 2 is:
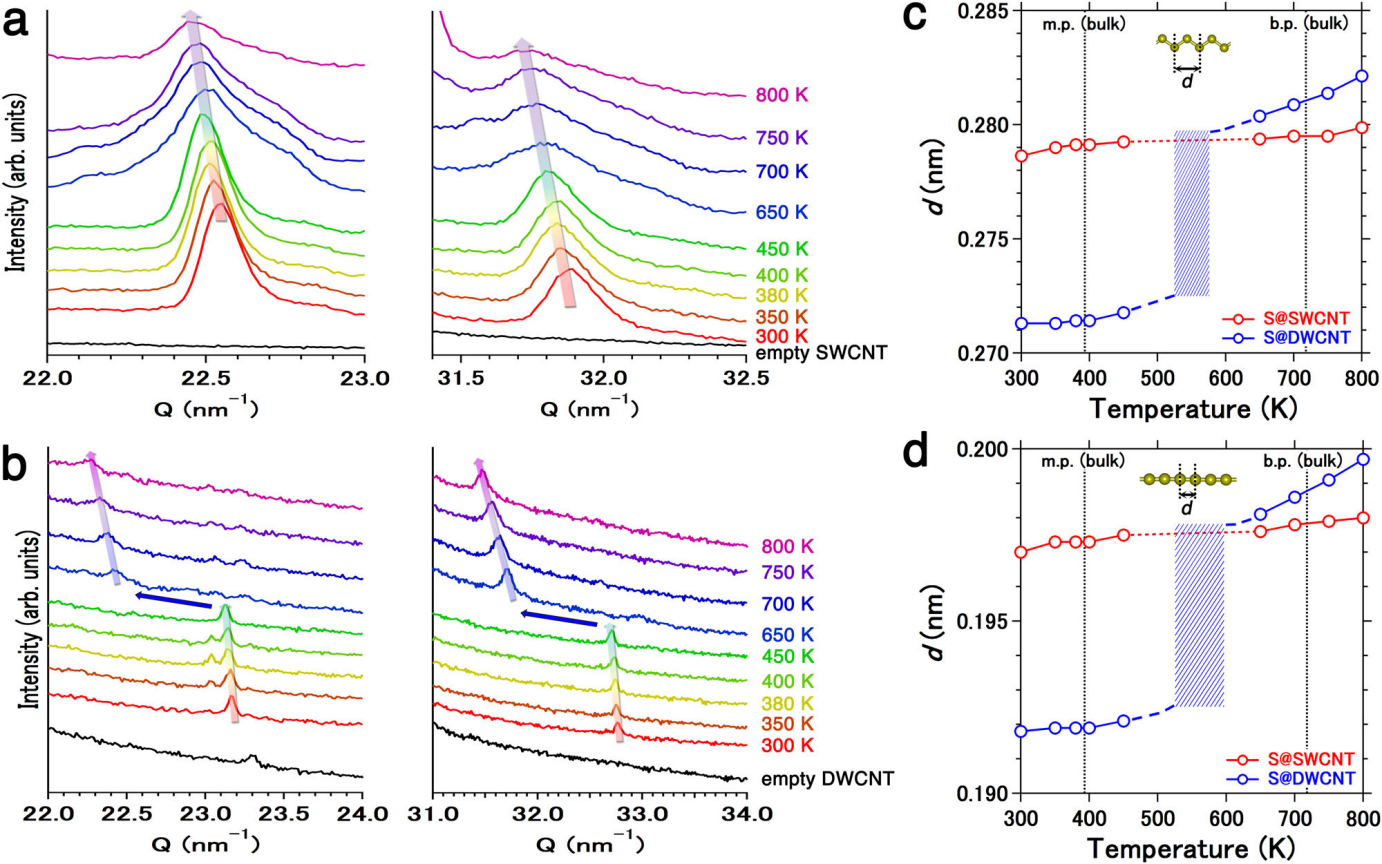


The error has not been corrected in the PDF or HTML versions of the Article.

