# Supplementary material for: Conducting linear chains of sulphur inside carbon nanotubes
Source: Nat Commun. 2013 Jul 12;4:2162. doi: 10.1038/ncomms3162 (PMC3717502; doi:10.1038/ncomms3162)
Supplement: Supplementary Information — Supplementary Figures S1-S15, Supplementary Tables S1-S2, Supplementary Notes 1-6 and Supplementary References [file ncomms3162-s1.pdf]

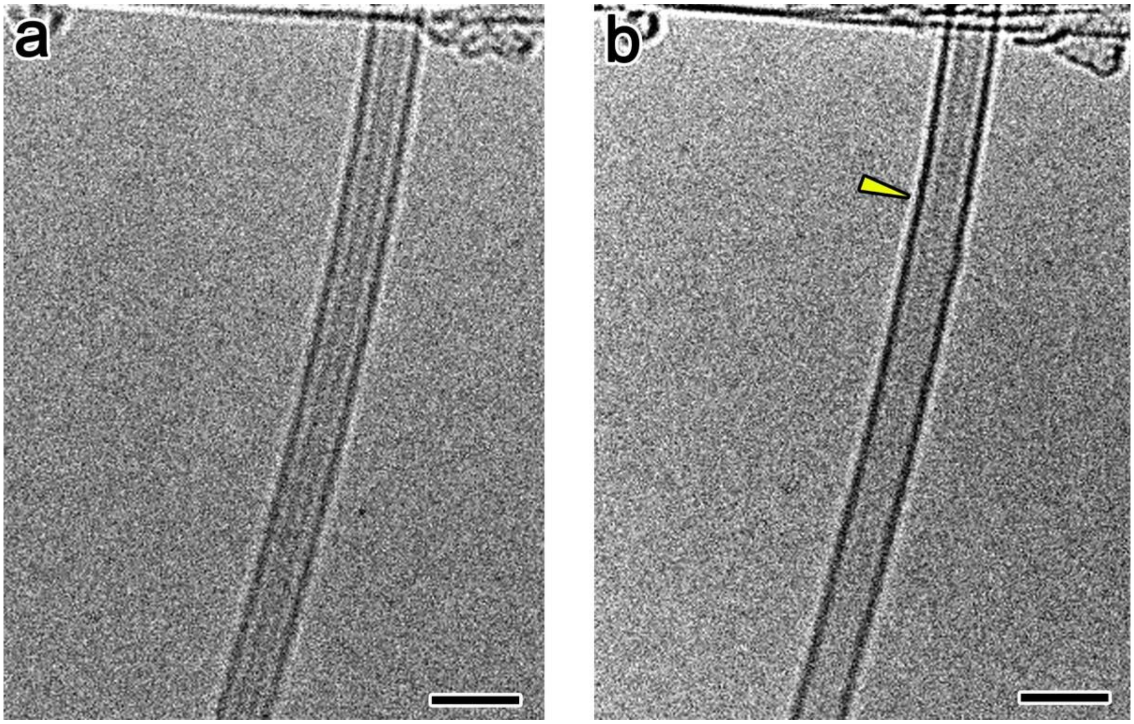

Supplementary Figure S1. Translational motion of the 1D sulfur chains inside an SWCNT. a, HRTEM image of S@SWCNT at the initial stage of the observation. The two lines, which can be ascribed to the 1D sulfur chains, are clearly visible across the entire length of the SWCNT. **b,** Subsequent HRTEM image, taken several tens of seconds after image (**a**). Visible line ends, marked by the arrow, suggest simultaneous translational motion of enclosed 1D sulfur chains. Scale bar, 2 nm. Owing to the fact that electron irradiation is unable to displace axially the entire inner wall of a DWCNT, we conclude that those two lines can be ascribed to the enclosed sulfur chains, not to the inner SWCNT. Since the two lines move simultaneously, we have reason to believe that the two 1D sulfur chains interact.

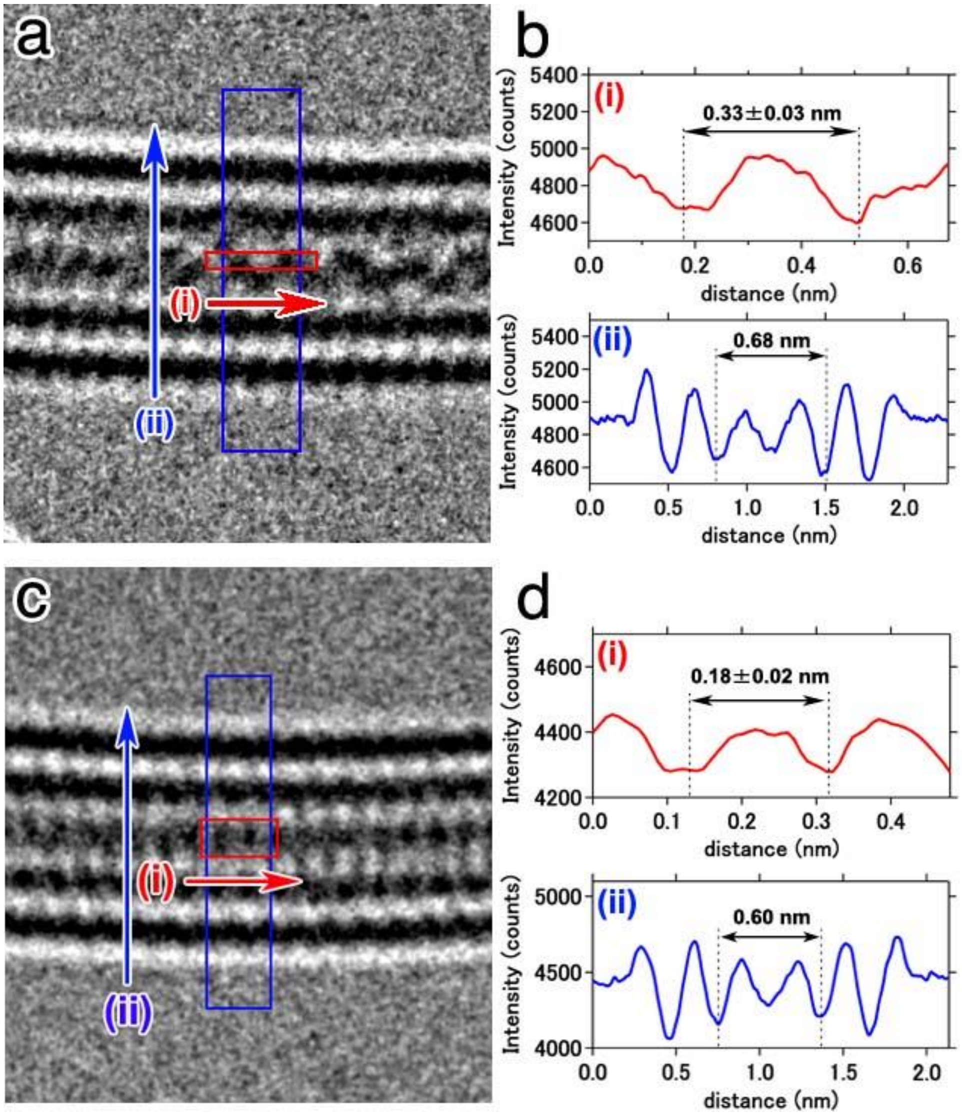

Supplementary Figure S2. HRTEM analysis of the 1D sulfur chains inside DWCNTs. a, HRTEM image of the 1D sulfur chain in zigzag conformation encapsulated in a DWCNT. **b,** Line profiles of the zigzag sulfur chain at positions (i) and (ii) along the red and blue arrows shown in (**a**). The TEM analysis of the zigzag sulfur chain encapsulated inside the inner CNT wall with 0.68 nm diameter indicates a lattice constant of 0.33±0.03 nm on the average, which is in agreement with data from the XRD analysis (d=0.271 nm). We note that these data describe a zigzag chain with two S atoms per unit cell. **c,** HRTEM image of the 1D sulfur chain in linear conformation encapsulated in a narrower DWCNT. **d,** The line profiles at position (i) and (ii) along the red and blue arrows shown in (**c**). In the case of the linear sulfur chain, which is encapsulated in the narrower inner CNT wall with 0.60 nm diameter, the primitive unit cell contains only one atom. The observed lattice constant of 0.18±0.02 nm agrees again very well with results of the the XRD analysis (d=0.192 nm). For the sake of fair comparison, the lattice constant of a linear 2-atom supercell is then 0.36±0.04 nm, somewhat larger than the 0.33 nm value in the zigzag nanotube.

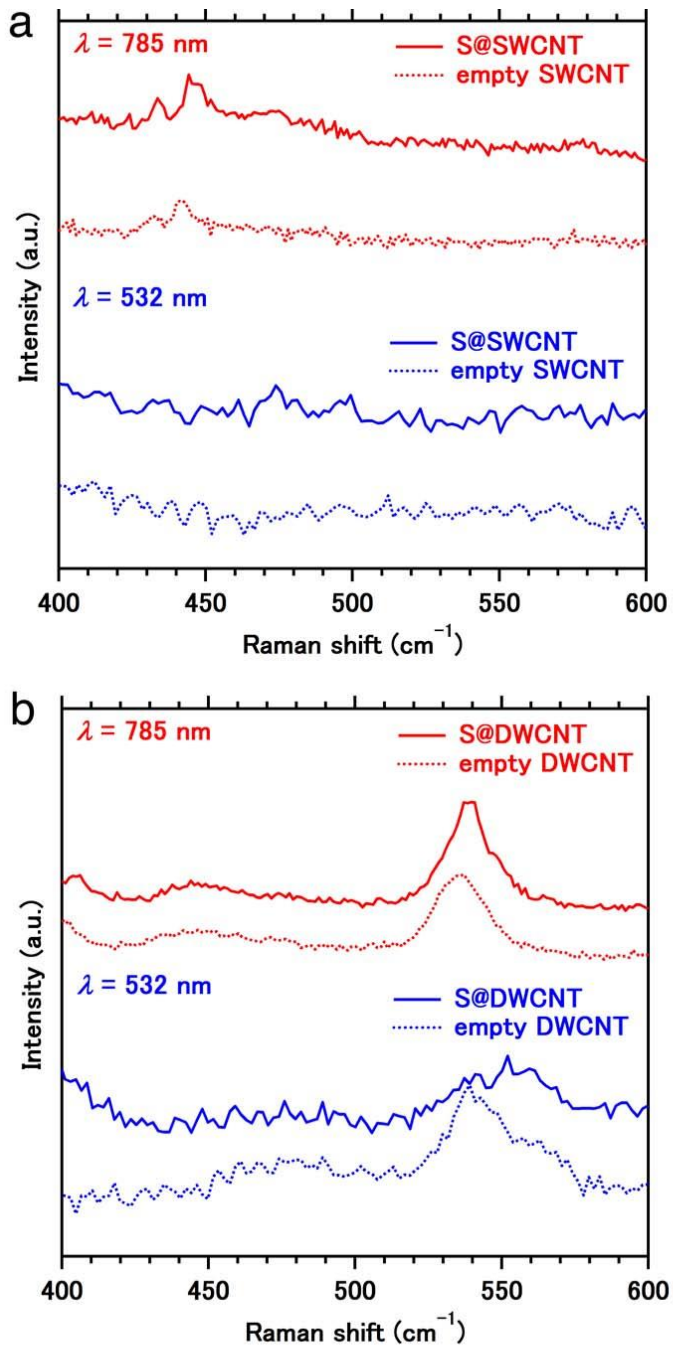

Supplementary Figure S3. Resonance Raman spectra in the frequency range of the S-S stretching modes. Resonance Raman spectra of (**a**) empty SWCNT and S@SWCNT, (**b**) empty DWCNT and S@DWCNT, obtained with 532 nm and 785 nm laser excitations. Supplementary Fig. S3 compares resonance Raman spectra of empty CNTs and S@CNTs, in the frequency range of 400-600 cm^{-1} where the S-S stretching modes are usually observed for bulk sulfur[30,51]. Despite the fact that 3D helical sulfur chains show strong Raman peaks around 450 cm^{-1}, this characteristic S-S stretching mode is obscured for S@SWCNT and S@DWCNT, indicating negligible amounts of 3D helical chains inside CNTs.

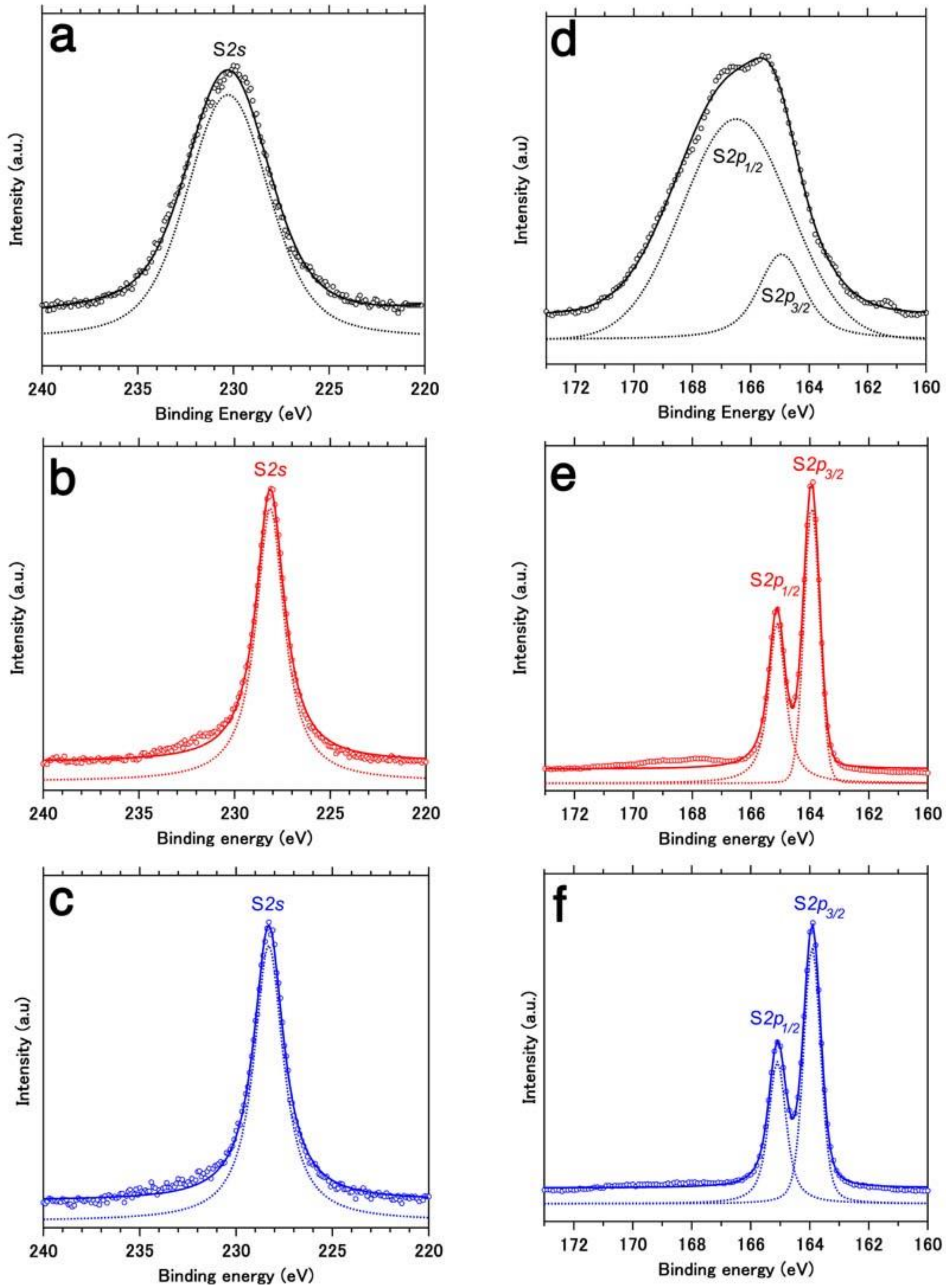

Supplementary Figure S4. XPS analysis at S*2s* and S*2p* core-level regions of 1D sulfur chains. Detailed analysis of the XPS spectra of the S*2s* (**a**-**c**) and the S*2p* (**d**-**f**) core-level regions in bulk sulfur (**a** and **d**), S@SWCNT (**b** and **e**), and S@DWCNT (**c** and **f**). Open circles are the experimental data, solid lines represent the Voigt function fit, and dotted lines show the components of the fitted curves. The binding energies are presented in Supplementary Table S1.

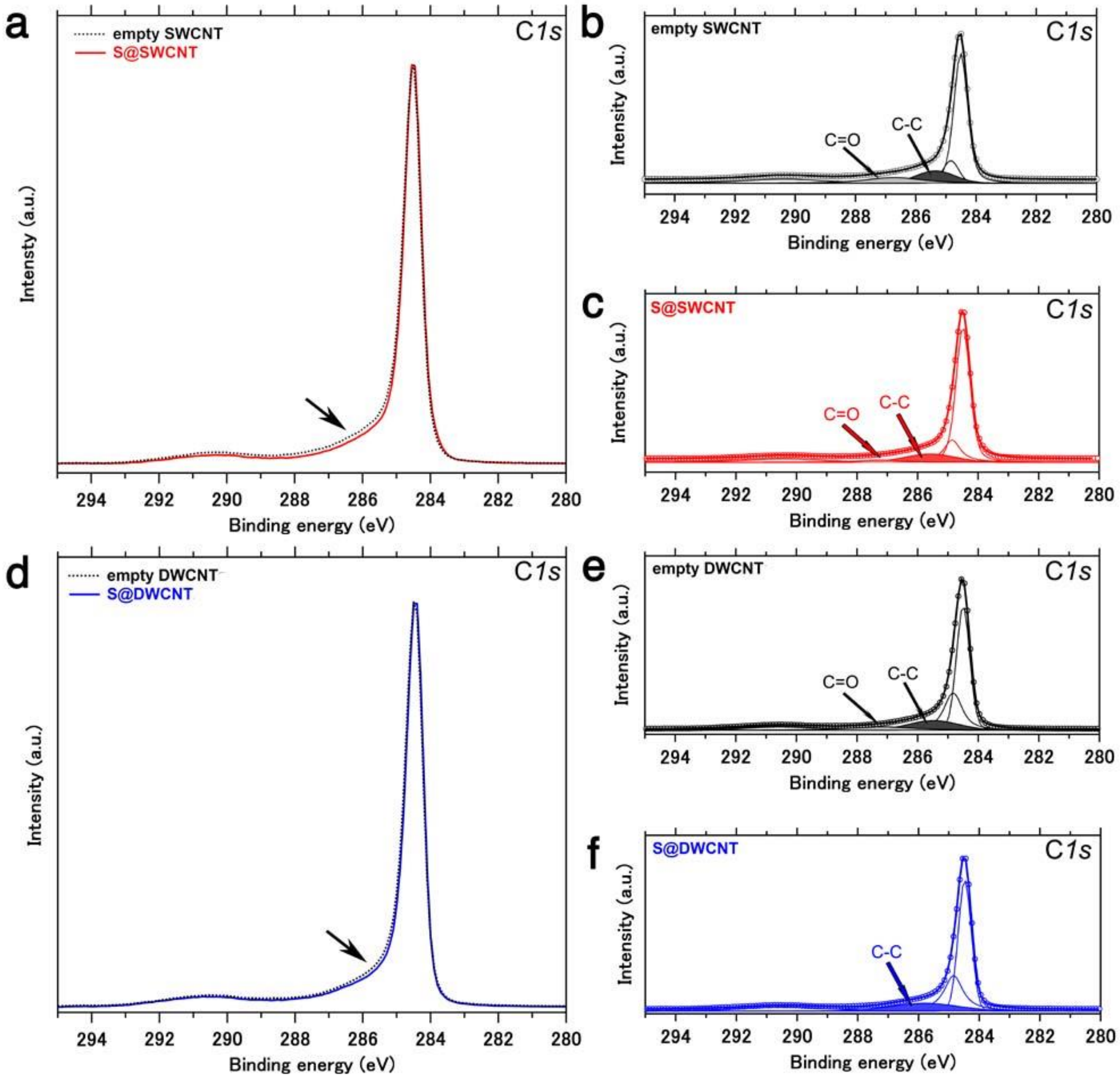

Supplementary Figure S5. XPS analysis at C*1s* core-level region of empty-CNTs and S@CNTs. XPS C*1s* core-level spectra of empty SWCNTs and S@SWCNTs (**a**) and those of empty DWCNTs and S@DWCNTs (**d**). Main changes in the XPS spectra are shown by the arrows. Deconvoluted XPS spectra of empty SWCNTs (**b**), S@SWCNTs (**c**), empty DWCNTs (**e**), and S@DWCNT (**f**).

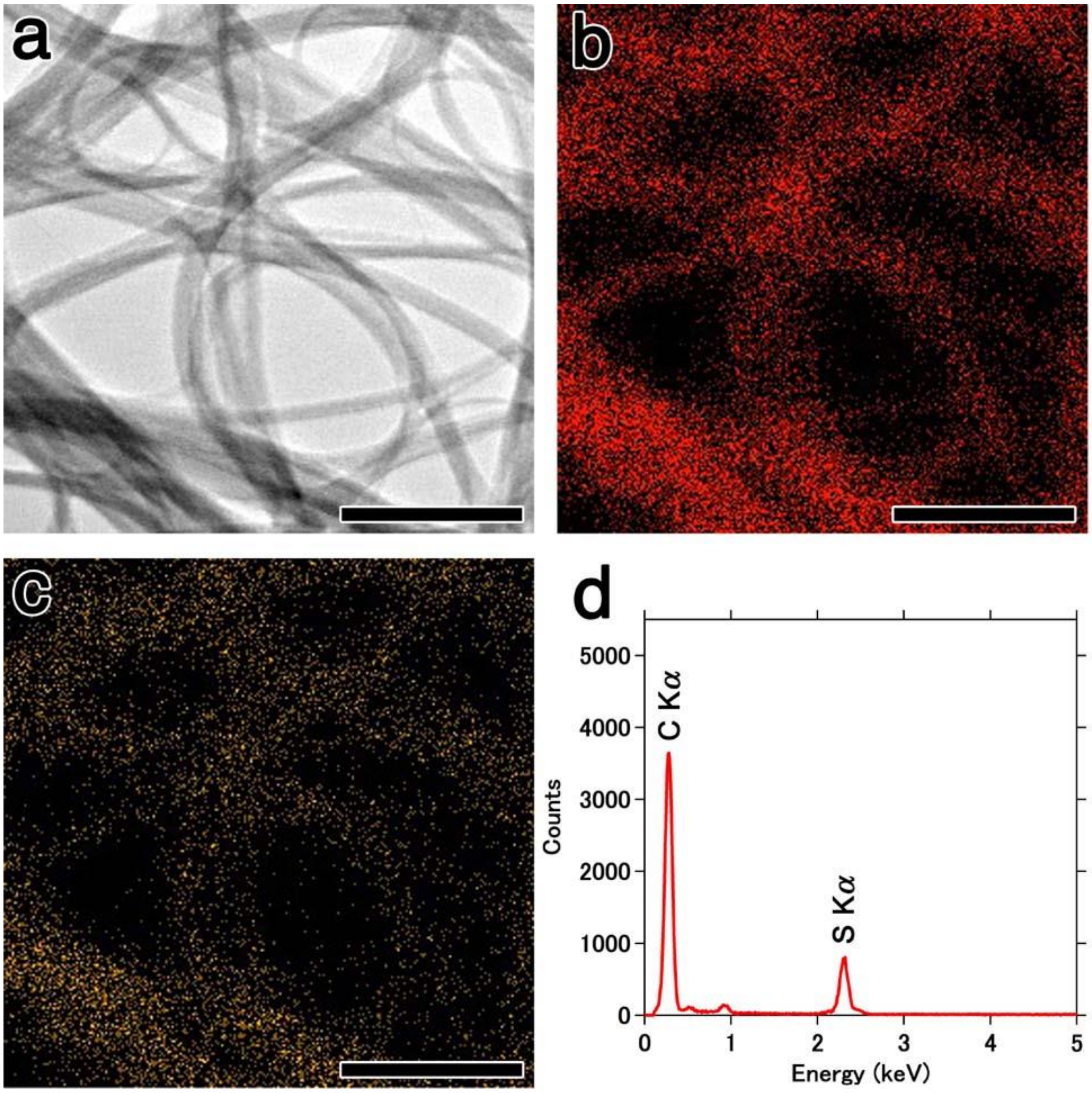

Supplementary Figure S6. Elemental mapping analysis of S@SWCNT. STEM image of S@SWCNT (**a**), elemental mapping of carbon (**b**) and sulfur (**c**). The EDS spectrum acquired during the integration across the entire STEM image (**d**). Scale bar, 200 nm.

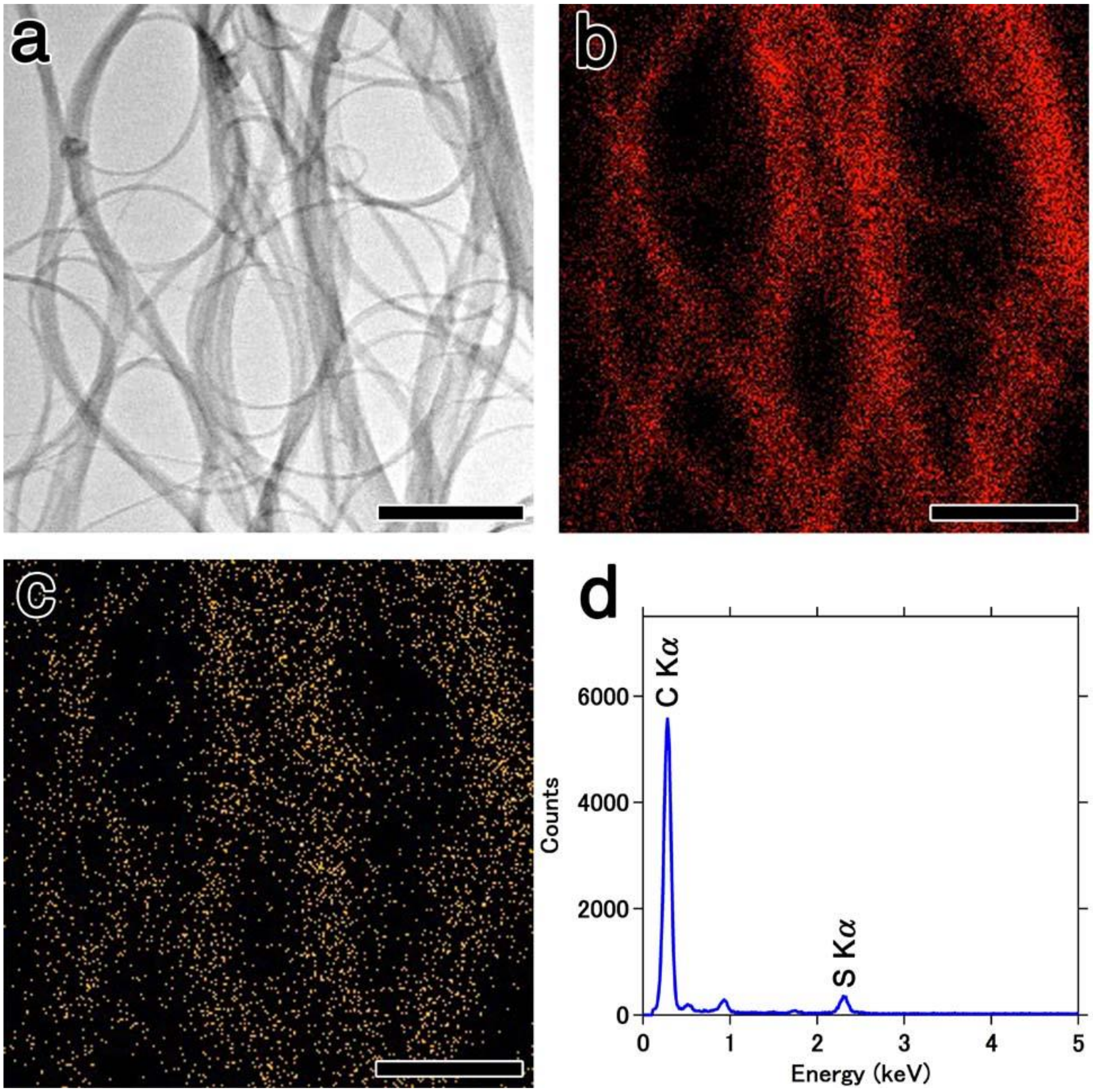

Supplementary Figure S7. Elemental mapping analysis of S@DWCNT. STEM image of S@DWCNT (**a**), elemental mapping of carbon (**b**) and sulfur (**c**). The EDS spectrum acquired during the integration across the entire STEM image (**d**). Scale bar, 200 nm.

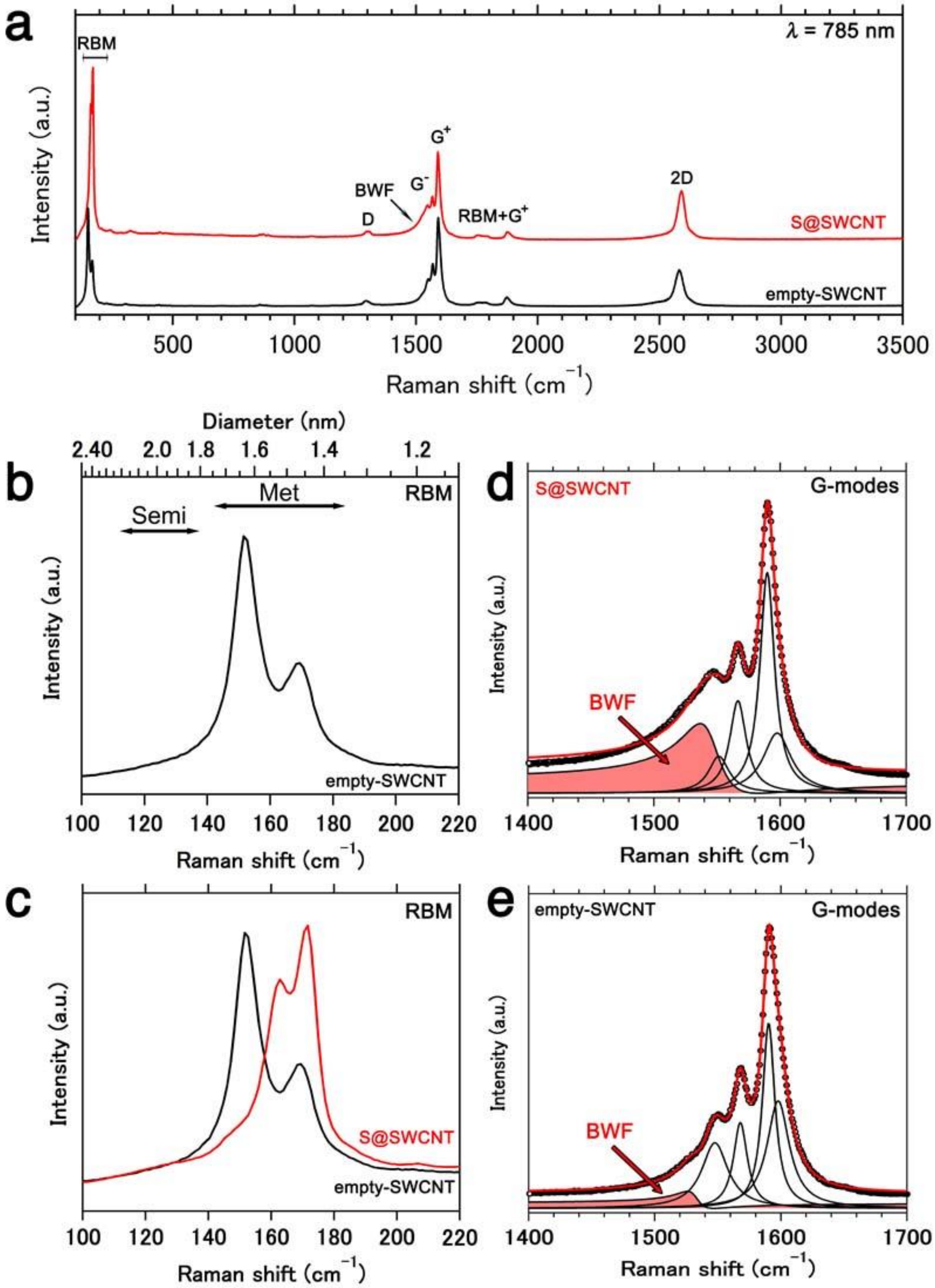

Supplementary Figure S8. Raman spectral analysis of empty-SWCNT and S@SWCNT obtained with the 785 nm excitation. a, Raman spectra of S@SWCNTs and empty SWCNTs obtained with the 785 nm excitation (100-3500 cm^{-1}). **b,** Raman spectrum of empty SWCNTs in the RBM region, indicating that metallic SWCNTs contribute most of the signal with 785 nm excitation. **c,** Comparison of RBM frequencies between S@SWCNTs and empty SWCNTs. Detailed analysis of the G-band region for (**d**) S@SWCNTs and for (**e**) empty SWCNTs.

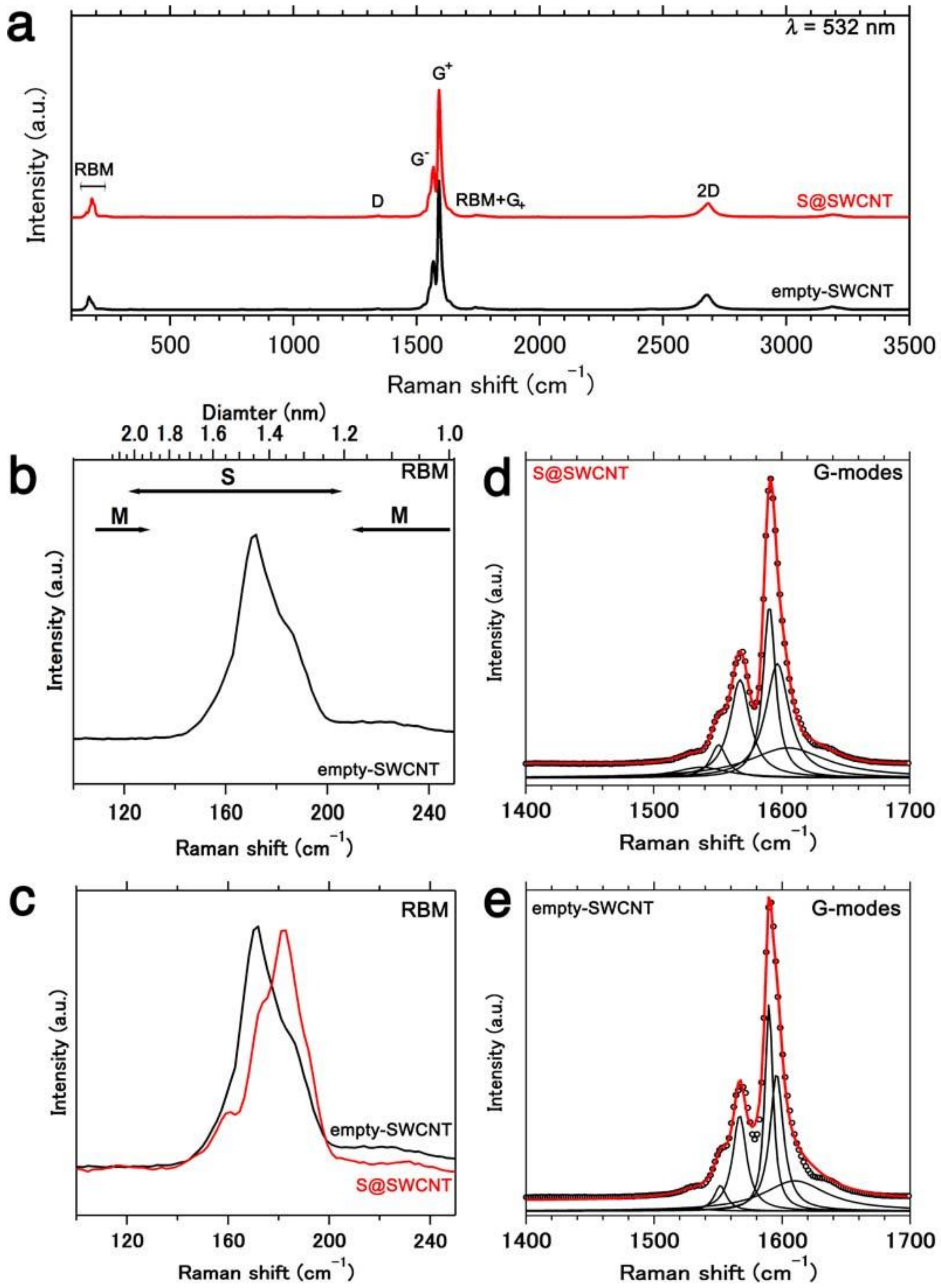

Supplementary Figure S9. Raman spectral analysis of empty-SWCNT and S@SWCNT obtained with the 532 nm excitation. a, Raman spectra of S@SWCNTs and empty SWCNTs obtained with the 532 nm excitation (100-3500 cm^{-1}). **b,** Raman spectrum of empty SWCNTs in the RBM region, indicating that semiconducting SWCNTs contribute most of the signal with the 785 nm excitation. **c,** Comparison of RBM frequencies between S@SWCNTs and empty SWCNTs. Detailed analysis of the G-band region for (**d**) S@SWCNTs and for (**e**) empty SWCNT.

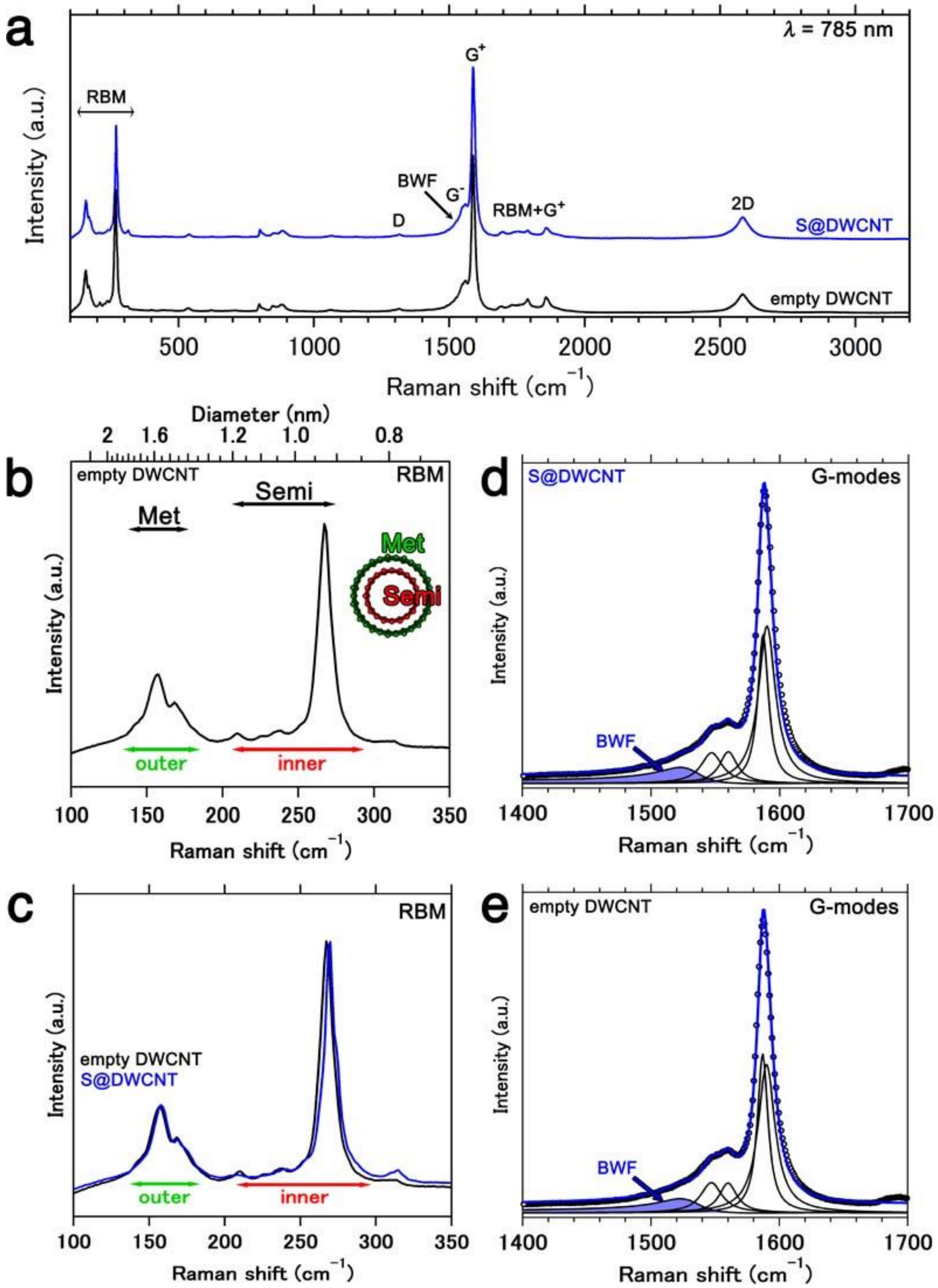

Supplementary Figure S10. Raman spectral analysis of empty-DWCNT and S@DWCNT obtained with the 785 nm excitation. a, Raman spectra of S@DWCNTs and empty DWCNTs obtained with the 785 nm excitation (100-3200 cm^{-1}). **b,** Raman spectrum of empty DWCNTs in the RBM region, indicating that metallic CNTs contribute most of the signal for outer CNTs with the 785 nm excitation. **c,** Comparison of RBM frequencies between S@DWCNTs and empty DWCNTs. Detailed analysis of the G-band region for (**d**) S@DWCNTs and for (**e**) empty SDWCNTs. Note that a BWF line is associated with the outer CNT.

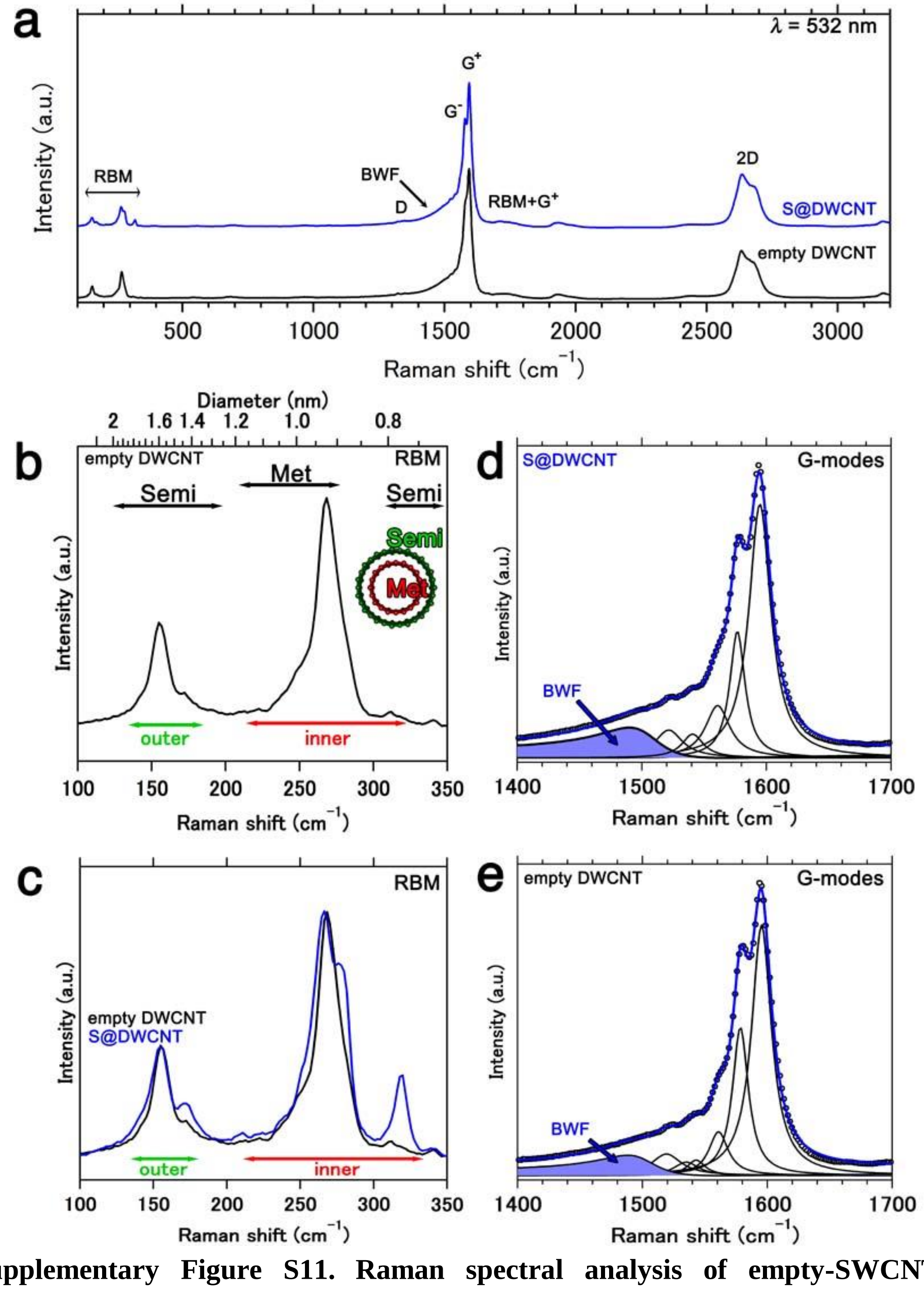

Supplementary Figure S11. Raman spectral analysis of empty-SWCNT and S@SWCNT obtained with the 532 nm excitation. a, Raman spectra of S@DWCNTs and empty DWCNTs obtained with the 532 nm excitation (100-3200 cm^{-1}). **b,** Raman spectrum of empty DWCNTs in the RBM region, indicating that metallic CNTs contribute most of the signal for inner CNTs with the 532 nm excitation. **c,** Comparison of RBM frequencies between S@DWCNTs and empty DWCNTs. Detailed analysis of the G-band region for (**d**) S@DWCNT and for (**e**) empty DWCNT. Note that a BWF line is associated with the inner CNT.

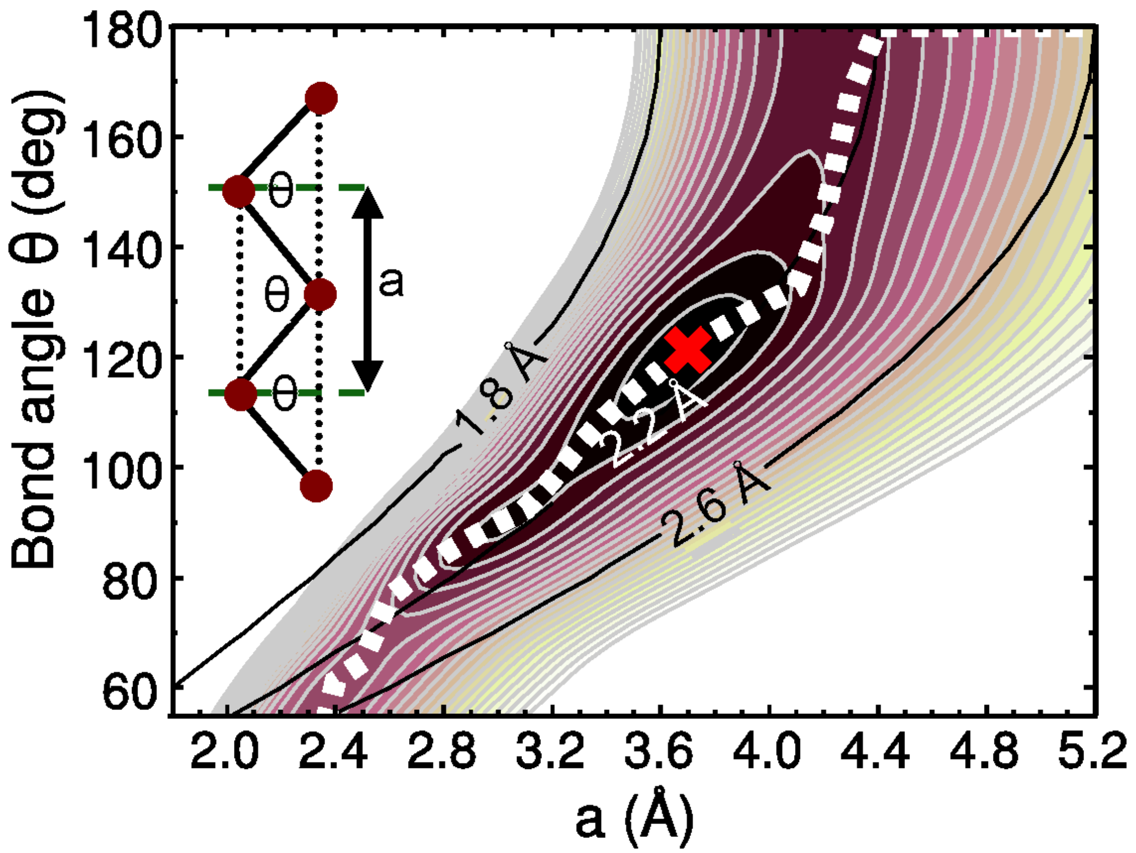

Supplementary Figure S12. Contour plot of the calculated total energy as a function of bond angle and unit length of an isolated sulfur chain. Energy of a neutral S chain as a function of the lattice constant a and the bond angle θ, adapted from Ref. (7). The global optimum is denoted by **x**. Contour lines, representing the total energy per 2-atom unit cell, are separated by 0.2 eV. Optimized geometries for each lattice constant are shown by the white dotted lines. Lines of constant bond length are indicated by black solid lines. The inset depicts schematically the unit cell of length a and the bond angle θ. The free-standing sulfur chain favors a planar zigzag structure with two atoms in the unit cell, shown schematically in the inset. The optimum free-standing structure, marked by **x,** has the lattice constant a_{eq}=3.6 Å and the bond angle θ_{eq}=120º.

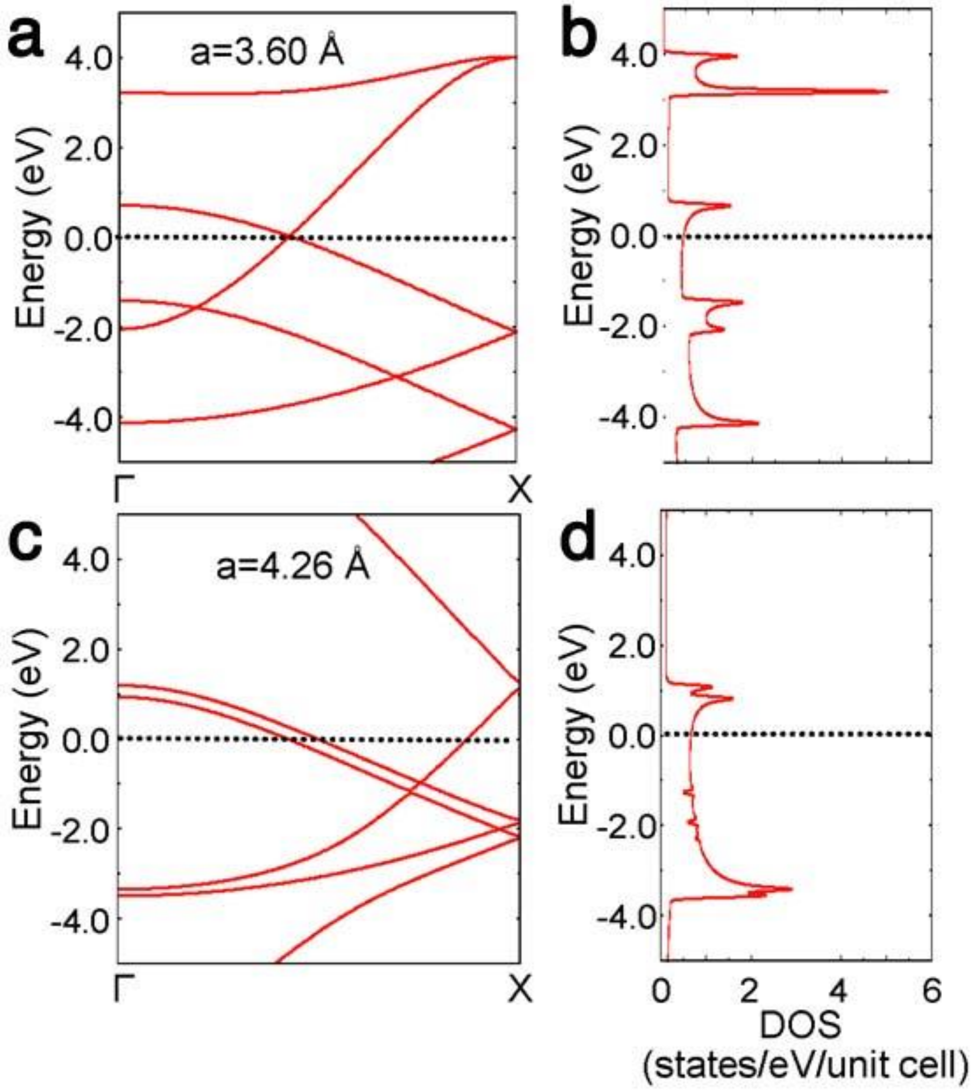

Supplementary Figure S13. Electronic structures of isolated sulfur chains. Electronic band structure (**a** and **c**) and density of states (**b** and **d**) of sulfur chains. Results in (**a**) and (**b**) for the optimum structure are compared to those in (**c**) and (**d**) for a stretched chain that is commensurate with the lattice constant of an (8,0) SWCNT. The Fermi level is set at E=0. The electronic structure of a free-standing sulfur chain, shown in Supplementary Figs. S13a and S13b, indicates that this system is metallic, with two bands with $3p_{\pi}$ character crossing the Fermi level. Depending on the precise position in the 1D Brillouin zone, where the valence bands cross E_F, a Peierls instability could open a band gap at E_F if the unit cell size were doubled. This electronic instability is suppressed by moving the band crossing away from half-way between Γ and X due to the steric constraints imposed by the surrounding nanotube, and by the small charge transfer between the sulfur chain and the nanotube. As shown in Supplementary Figs. S13c and S13d, stretching the chain decreases the overall bandwidth and moves the two valence bands closer together. Whereas E_{F} is crossed by two bands in Supplementary Fig. S13a, it is crossed by three bands in Supplementary Fig. S13c, thus increasing the density of states near E_{F} and consequently the conductance of the stretched chain.

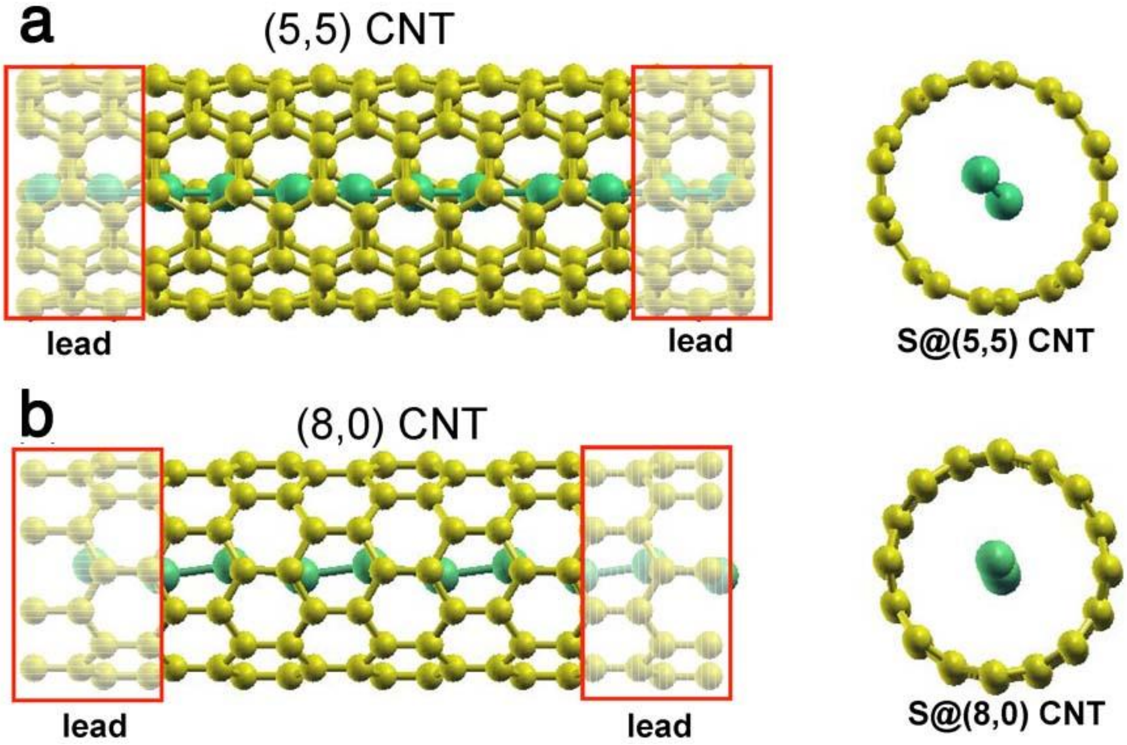

Supplementary Figure S14. Optimized model structures of sulfur chains inside a SWCNT. Side view (left panels) and end-on view (right panels) of sulfur chains enclosed inside (**a**) a (5,5) armchair and (**b**) an (8,0) zigzag SWCNTs. The (5,5) and (8,0) SWCNTs have a near-optimum diameter to enclose sulfur chains.

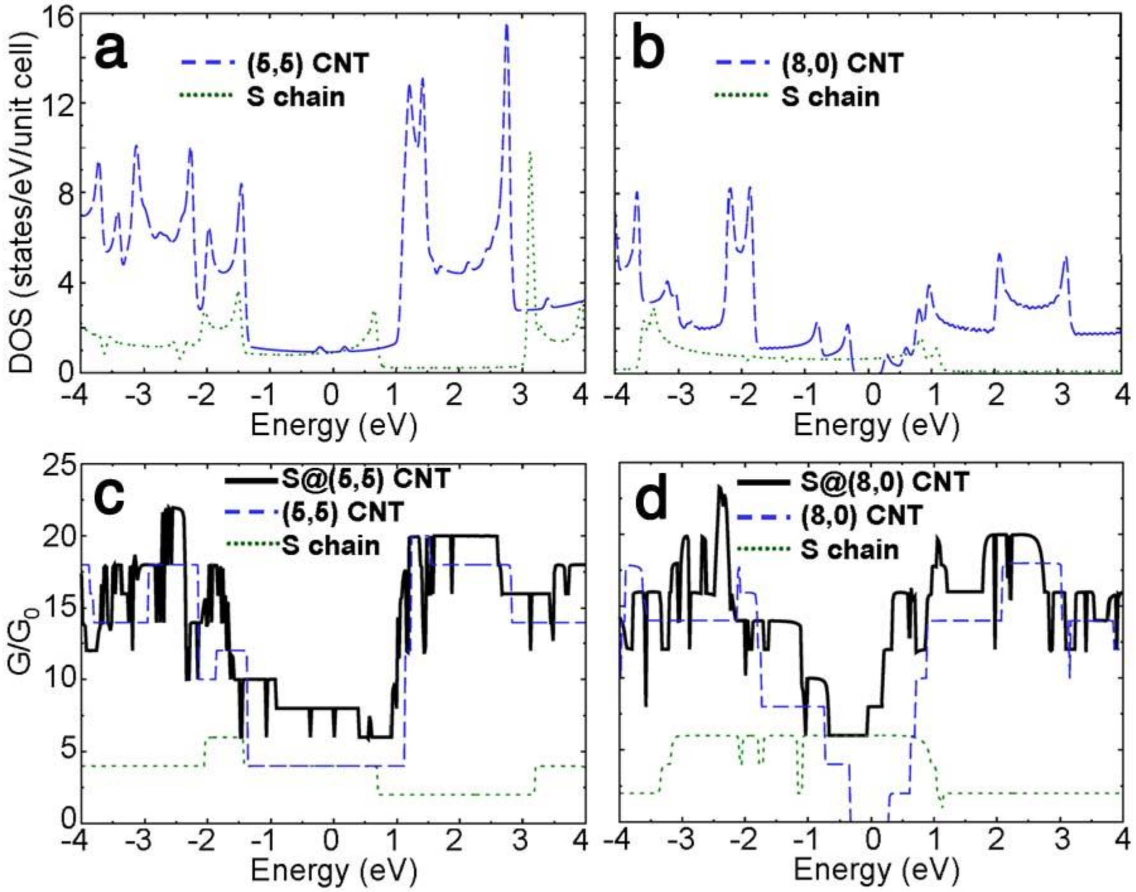

Supplementary Figure S15. Electronic structures and quantum conductance of sulfur chains inside SWCNTs. The electronic density of states of (**a**) a metallic (5,5) and (**b**) a semiconducting (8,0) SWCNT, shown by the blue dashed line. The electronic density of states of an isolated sulfur chain with lattice constants commensurate with those of the (**a**) (5,5) and (**b**) (8,0) SWCNTs, shown by the green dotted line. Ballistic quantum conductance of a sulfur chain enclosed inside (**c**) a (5,5) SWCNT and (**d**) an (8,0) SWCNT, shown by the heavy solid black lines. Corresponding quantum conductance results for isolated SWCNTs are shown by the blue dashed lines and those of isolated sulfur chains in the proper geometry by the green dotted lines. E=0 denotes the Fermi level in (**a**) and (**b**) and the carrier injection energy in a neutral, unbiased system in (**c**) and (**d**). G_0 is the conductance quantum. Assuming that the enclosed sulfur chain would not interact electronically with the enclosing nanotube, the total conductance of the S@SWCNT system should be a superposition of the quantum conductance of the isolated chain and the isolated SWCNT. The quantum conductance of both system components is plotted in Supplementary Fig. S15 along with the conductance of S@SWCNT. In each case, as indicated in Supplementary Fig. S14, we used the same system to describe the leads and the scattering region. Generally, we find the quantum conductance of the S@SWCNT composite system to be very close to the superposition of the components, with the exception of dips in the conductance curve, characteristic of the small S—SWCNT interaction.

Supplementary Table S1. The S*2s* and S*2p* core-level binding energies of bulk sulfur and S@CNTs

Sample	Binding energy (eV)		
	S2s	$S2p_{1/2}$	$S2p_{3/2}$
Bulk sulfur (α-S_8)	230.3	166.5	165.0
S@SWCNTs	228.1	165.1	163.9
S@DWCNTs	228.3	165.1	163.9

Supplementary Table S2. Frequencies of the G^+ and the 2D bands (cm^{-1}), obtained with λ=785 and 532 nm laser excitations.

Sample	λ=785 nm		λ=532 nm	
	G^+	2D	G^+	2D
Empty SWCNT	1591	2581	1591	2677
S@SWCNT	1590	2590	1591	2683
Empty DWCNT	1587	2584	1594	2632, 2679
S@DWCNT	1588	2584	1594	2634, 2683

Supplementary Note 1. XPS analysis at S*2s* and S*2p* core-level regions

Supplementary Table S1 shows the S*2s* and S*2p* core-level binding energies analyzed from the XPS spectra of bulk sulfur (α-S_8; the most stable, ring-shaped sulfur allotrope), S@SWCNTs, and S@DWCNTs shown in Figs. 3a and 3b of the main article and in Supplementary Fig. S4. The binding energies of the S*2p* core-level are split into two components (S$2p_{1/2}$ and S$2p_{3/2}$) arising from spin-orbit coupling. In order to obtain an accurate XPS spectrum of the insulating bulk sulfur sample, Au powder was mixed with a powder of bulk sulfur before the XPS measurements, ensuring a good electrical conduction in the XPS equipment. Calibration for the XPS spectra of bulk sulfur was performed using Au$4f_{5/2}$ and Au$4f_{7/2}$ binding energies of 87.5 eV and 83.8 eV, respectively[52]. No Au powder was used when recording the XPS spectra of S@SWCNTs and S@DWCNTs. To evaluate the binding energies, the measured XPS spectra were fitted with the Voigt function after subtracting the background signal using the commonly-used Shirley equation (Supplementary Fig. S4).

Supplementary Note 2. XPS analysis at C*1s* core-level region

Supplementary Fig. S5 shows the XPS C*1s* core-level spectra of empty CNTs and S@CNTs. The main peak at 284.5 eV is assigned to the binding energy of the sp^2 bonded carbon[53] in empty SWCNTs and empty DWCNTs (Supplementary Figs. S5a and S5d). Whereas the binding energy does not change after the filling with 1D sulfur chains, the intensities of the shoulder peaks around 286 eV are slightly weakened in both filled SWCNTs and DWCNTs, as marked by arrows in Supplementary Figs. S5a and S5d. These changes can be traced back to the desorption of functional groups attached to the surface of pristine CNTs during the encapsulation process, which involve the vaporization of sulfur and expose the CNTs to high temperature of 873 K. Supplementary Figs. S5b and S5c display the deconvoluted XPS spectra of empty SWCNT and S@SWCNT, respectively, fitted using the Voigt function. The corresponding spectra of empty DWCNT and S@DWCNT are presented in Supplementary Figs S5e and S5f, respectively. Detailed analysis of the deconvoluted spectra reveals that the weakening of the shoulder peaks originates in the desorption of C=O groups (287.0 eV) and functional groups containing C-C single bonds (285.5 eV)[53]. Those results indicate that the slight changes in the XPS C*1s* core-level spectra of both the SWCNT and DWCNT systems are not caused by charge transfer involving the 1D sulfur chains. Consequently, we conclude that the decreases in electric resistivity following the filling by sulfur, depicted in Fig. 5 of the main text, is the consequence of additional electron transmission channels associated with 1D chains of metallic sulfur encapsulated in CNTs.

Supplementary Note 3. Elemental mapping analysis

Supplementary Fig. S6a shows an STEM image of S@SWCNT. Elemental mapping of carbon and sulfur acquired at the same location in the STEM image are depicted in Supplementary Figs. S6b and S6c, respectively. Supplementary Fig. S6d shows the EDS spectrum acquired during the integration across the entire STEM image. Our elemental mapping results indicate that carbon and sulfur are the main components of S@SWCNT. The elemental mapping analysis reveals that the locations of carbon and sulfur overlap uniformly throughout the image, indicating the high filling ratio of sulfur inside

SWCNTs. The elemental mapping analysis of S@DWCNT also confirms uniform encapsulation of sulfur inside DWCNTs (Supplementary Fig. S7).

Supplementary Note 4. Raman spectral analysis of RBM and BWF line

Supplementary Fig. S8a shows Raman spectra of S@SWCNT and empty SWCNT, measured with the 785 nm excitation. The sharp peaks in the low frequency region are so-called radial breathing modes (RBM). The RBM frequency, ω_{RBM}, follows a relation ω_{RBM}=248 cm^{-1}/d_t, where d_t is tube diameter in nanometers[32]. According to the Kataura plot[36], the tube diameter is directly related to the energy gap between the van Hove singularities in the valence and conduction bands of CNTs. This establishes a selective probe of semiconducting or metallic CNTs in particular resonance conditions for incoming laser light. RBM analysis reveals that metallic SWCNTs are the main components when excited with 785 nm laser beam (Supplementary Fig. S8b). Another important feature of the RBM is the blue shift caused by material encapsulated in the CNT cavity[54]. Supplementary Fig. S8c explicitly shows the blue shift of the RBMs, indicating efficient encapsulation of the 1D sulfur chains. The Raman spectra of S@SWCNT and empty SWCNT are deconvoluted into four components, fitted by Lorentzian functions, and one Breit-Wigner-Fano (BWF) line (Supplementary Figs. S8d and S8e). The BWF line shape is given by

$$I(\omega) = I_0 \{1+(\omega-\omega_0) / q\Gamma\} / \{1+((\omega-\omega_0) / \Gamma)^2\}, \quad \text{(S1)}$$

where I_0, ω_0, q, Γ are intensity, renormalized frequency, lineshape parameter and broadening parameter, respectively[36]. In comparison with the BWF line of empty SWCNT (Supplementary Fig. S8e), the BWF intensity is significantly enhanced following the encapsulation of 1D sulfur chains (Supplementary Fig S8d), as also depicted in Fig. 4a. Detailed explanation is given in the main text.

Supplementary Fig. S9a shows Raman spectra of S@SWCNTs and empty SWCNTs, measured with the 532 nm excitation. Here, semiconducting SWCNTs are the dominant components in the Raman spectra with the 532 nm excitation (Supplementary Fig. S9b). Supplementary Fig. S9c indicates encapsulation of sulfur in semiconducting SWCNTs. When the semiconducting SWCNT is in the resonance condition, changes in the G-mode region are obscured (Supplementary Figs. S9d and S9e), including the effect of encapsulation. This result implies that sulfur cannot provide conduction electrons for semiconducting SWCNTs.

Our RBM and G-mode analysis indicates that trends similar to the SWCNT system also occur in S@DWCNTs and empty DWCNTs (Supplementary Figs. S10 and S11). There are four possibilities to compose a DWNT from metallic or semiconducting SWNTs. These are Semi@Met, Met@Semi, Semi@Semi, and Met@Met, where Semi and Met represent semiconducting SWCNTs and metallic SWCNTs, respectively (*e.g.*, Semi@Met. describes a semiconducting SWCNT contained inside a metallic SWCNT). Our experiments using 532 nm and 785 nm laser lines suggest that Semi@Met (Supplementary Fig. S10b) and Met@Semi (Supplementary Fig. S11b) dominate in the sample. An important observation is that the BWF line is enhanced, when the 1D sulfur chains are enclosed in the Met@Semi DWCNT (Supplementary Fig. S11d). In this case,

we can conclude that the electronic interaction between the conducting 1D chain of sulfur and the enclosing metallic CNT is important for the net conductance of the system.

Supplementary Note 5. Negligible charge transfer by Raman spectral analysis

The G^+-mode frequency is sensitive to charge transfer in the CNT system, since electron doping causes bond softening and hole doping causes bond hardening. Such a charge transfer could occur between the enclosed structure and the enclosing nanotube. G^+-mode frequencies in empty SWCNTs, S@SWCNTs, empty DWCNTs, and S@DWCNTs, measured using the 785 and 532 nm laser excitations, are summarized in Supplementary Table S2. Since changes in the G^+-mode frequencies caused by encapsulation of sulfur are nearly negligible, we conclude that only very small charge transfer may occur between the 1D sulfur chains and CNTs, in agreement with the XPS analysis. Also our Mulliken population analysis reveals only a very small transfer of ≈0.1 electrons/S atom between the sulfur chains and the host CNTs, which is not expected to change the transport properties of S@SWCNT and S@DWCNT.

The 2D-mode, an overtone mode of the defect-induced mode (D-mode), is sensitive to charge transfer[37], but also to presence of structural constraints caused by encapsulation. The 2D-mode frequency of S@SWCNTs shows consistently blue shifts of 9 cm^{-1} and 6 cm^{-1} for 785 nm and 532 nm laser excitations, respectively (Supplementary Table S2). For S@DWCNTs, the frequency shift of the 2D-mode is negligible when probed by the 785 nm laser excitation, whereas blue shifts of 2-4 cm^{-1} are observed when probed by the 532 nm laser excitation.

Supplementary Note 6. Structural and electronic properties of sulfur chains encapsulated in carbon nanotubes

Since our interest is focused on chains enclosed inside CNTs, we selected the (5,5) armchair SWCNT with 0.68 nm diameter and the (8,0) zigzag SWCNT with 0.63 nm diameter as model enclosures. Energetically, these SWCNT diameters are near the optimum value to contain a sulfur chain, since the optimum distance between a sulfur chain and a graphene monolayer is 0.33 nm. At this distance, there is only a very small interaction between the encapsulated chain and the SWCNT. Since the Pauling electronegativity[31] of S and C have nearly the same value 2.5, we expect only a small charge transfer between S and C. This is confirmed by our single-ζ Mulliken population analysis that indicates a typical transfer of only ≈0.1 electrons per sulfur atom between the enclosed chain and the surrounding CNT. Our total energy calculations of the composite system S@CNT and its isolated, frozen components indicate a very small encapsulation energy of 0.20 eV/S atom for S@(5,5) and 0.24 eV/S atom for S@(8,0). The optimized structure of the sulfur chain inside these nanotubes is shown in Supplementary Fig. S14. For energy reasons, we do not expect filling of nanotubes with inner diameter below 0.7 nm by sulfur chains.

As expected and confirmed by the calculated electronic density of states of these free-standing SWCNTs, shown in Supplementary Figs. S15a and S15b, the (5,5) SWCNT is metallic, whereas the (8,0) SWCNT is semiconducting.

Supplementary References

51. Steudel, R. Properties of Sulfur-Sulfur Bonds. *Angew. Chem. Int. Ed.* **14**, 655-720 (1975).
52. Ikeo, N. *et al. Handbook of X-ray Photoelectron Spectroscopy* (JEOL, 1991).
53. Utsumi, S. *et al.* Direct Evidence on C-C Single Bonding in Single-Wall Carbon Nanohorn Aggregates. *J. Phys. Chem. C* **111**, 5572 (2007).
54. Bandow, S. *et al.* Smallest limit of tube diameters for encasing of particular fullerenes determined by radial breathing mode Raman scattering. *Chem. Phys. Lett.* **347**, 23-28 (2001).
